# Quantifying Covalency and Environmental Effects in RASSCF-Simulated O K-Edge XANES of Uranyl

**DOI:** 10.1021/acs.inorgchem.4c02144

**Published:** 2024-08-02

**Authors:** Kurtis Stanistreet-Welsh, Andrew Kerridge

**Affiliations:** Department of Chemistry, Lancaster University, Lancaster, LA1 4YB, U.K.

## Abstract

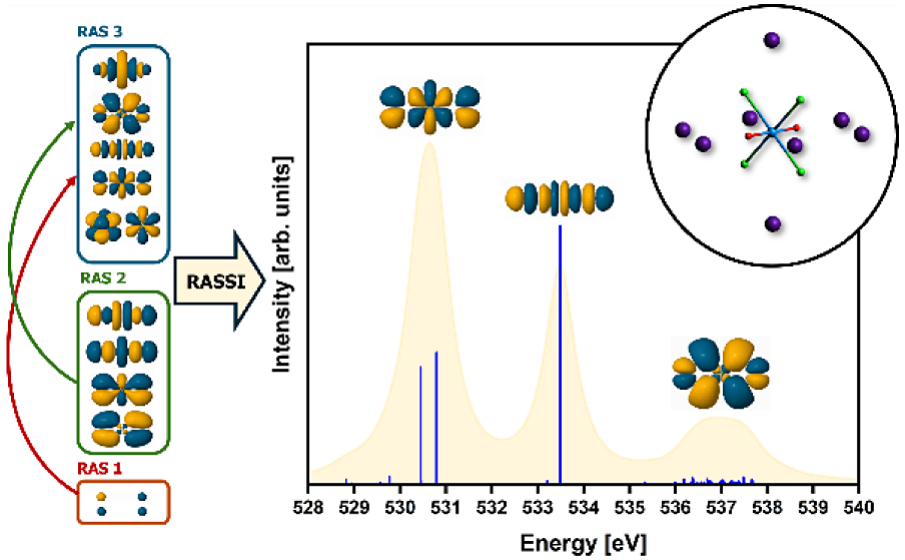

A RASSCF approach to simulate the O K-edge XANES spectra of uranyl is employed, utilizing three models that progressively improve the representation of the local crystal environment. Simulations successfully reproduce the observed three-peak profile of the experimental spectrum and confirm peak assignments made by Denning. The [UO_2_Cl_4_]^2–^ model offers the best agreement with experiment, with peak positions (to within 1 eV) and relative peak separations accurately reproduced. Establishing a direct link between a specific electronic transition and peak intensity is complicated, as a large number of possible transitions can contribute to the overall peak profile. Furthermore, a relationship between oxygen character in the antibonding orbital and the strength of the transition breaks down when using a variety of orbital composition approaches at larger excitation energy. Covalency analysis of the U–O bond in both the ground- and excited-state reveals a dependence on the crystal environment. Orbital composition analysis reveals an underestimation of the uranium contribution to ground-state bonding orbitals when probing O K-edge core-excited states, regardless of the uranyl model employed. However, improving the environmental model provides core-excited state electronic structures that are better representative of that of the ground-state, validating their use in the determination of covalency and bonding.

## Abstract 2

A RASSCF approach to simulate the O K-edge XANES spectra of uranyl is employed, utilizing three models that progressively improve the representation of the local crystal environment. Simulations successfully reproduce the observed three-peak profile of the experimental spectrum and confirm peak assignments made by Denning.

## Introduction

Developing a fundamental understanding of covalency in actinide compounds is key to exploiting their unique properties in various applications, ranging from the development of new magnets^1−3^ and catalysts,^4−6^ to the separation of spent nuclear fuels.^7−9^ In particular, understanding the differences in covalency between minor actinides and lanthanides allows for the design of improved ligands capable of separating these species in high level nuclear waste.^8,9^ Improvements in spent nuclear fuel separations could enable better strategies for long-term storage and recycling of nuclear waste, improving the prospects of using nuclear energy as an alternative to fossil fuel intensive power generation. Practically, the characterization of actinide bonding can be difficult, as actinide systems exhibit pronounced relativistic effects, weak crystal fields, strong electron correlation, and substantial multiconfigurational character. Therefore, modeling covalency in these systems also poses a unique challenge for simulations. Covalency, as proposed by Heitler and London,^10^ can be considered within perturbation theory as a deviation away from the ionic limit,^11−14^ manifesting due to the near-degeneracy and/or spatial overlap of atomic orbitals. Of particular interest is the involvement of actinide 5f- and 6d-orbitals in bonding, with other studies suggesting that the 6p, 7s and 7p orbitals may also play a significant role.^11−13,15−22^ For example, the higher σ_u_ orbital energy relative to the other valence orbitals in uranyl can be explained through a “pushing from below” mechanism, whereby the uranium 6p admixes with the 5f and oxygen 2p combinations.^17,23,24^

X-ray absorption near-edge spectroscopy (XANES) paired with theoretical simulations has emerged as a powerful tool for probing metal–ligand covalency. Hedman, Solomon and coworkers pioneered the use of ligand K-edge XANES as a probe of covalency within transition metal complexes.^25−29^ Subsequent studies have since then explored the use of ligand K-edge as a tool for exploring covalency in a number of different actinide systems.^13−17,19,20,30−35^ Ligand K-edge XANES is particularly useful for probing the valence space of actinides, as peaks are generated by electric-dipole allowed core-excitations from ligand 1s orbitals into antibonding valence orbitals. The peak intensity is primarily driven by the degree of ligand p-orbital character in the latter, which by orthogonality arguments allows the quantification of ligand p-orbital contribution to the bonding orbitals.^14,19,28,35−37^ K-edge XANES therefore acts as a probe of ligand contributions to the bonding orbitals in the core-excited states (CESs). The established interpretation assumes that the mixing parameters determined for bonding orbitals in the CESs reflect those of the ground-state (GS). However, bonding orbitals may undergo substantial relaxation in response to the ligand core-hole and excited core–electron, rendering this assumption questionable.

Restricted Active-Space Self-Consistent Field (RASSCF) theory^38−41^ simulations have recently been utilized to successfully simulate the XANES spectra of several actinide systems.^14,30−33,35,42^ RAS approaches facilitate the treatment of multiconfigurational states and allow for orbital relaxation of the orbitals in the presence of the core-hole, allowing variation in covalency between the GS and CESs to be quantitatively investigated. In 2018, Autschbach and co-workers simulated the M_4/5_-edge XANES for actinyls ranging from uranyl to plutonyl.^32^ Here it was shown that the π-bonding orbitals in the GS and CESs are comparable, but metal contribution was underestimated in σ-bonding orbitals for neptunyl and plutonyl. We have also recently simulated the M_4/5_-edge XANES of uranyl and neptunyl using RAS approaches, as well as the O K-edge XANES for the same systems.^42^ We reported similar findings for the M_4/5_-edge CES bonding orbitals to that of Autschbach and co-workers, namely that they are comparable to the GS in uranyl, thus validating metal M_4/5_-edge XANES as a probe of GS uranyl covalency. However, O K-edge CES bonding orbitals were less reflective of those of the GS for uranyl, with GS uranium contributions underestimated by up to 10% when probing the CESs. This suggests that the validity of XANES as a valid probe of GS covalency is likely to vary depending on the nature of the system and the particular orbitals being probed.

In this contribution, the oxygen K-edge of uranyl is simulated using a multiconfigurational RASSCF approach and compared to the experimental work of Denning et al.^19^ The covalency of uranyl has been extensively studied due its prevalence as a structural motif in a number of hexavalent uranium complexes.^11,18,19,43,44^ A variety of equatorial ligands can bond with uranyl through largely electrostatic interactions.^43−47^ These ligands have been shown to influence uranyl covalency, for example, the longer and therefore weaker U–O bond in [UO_2_F_4_]^2–^ compared to [UO_2_Cl_4_]^2–^ is explained by the greater electrostatic repulsion between U and O in the presence of the fluoride ligands, which form shorter bonds with uranium compared to chloride.^44,45^ Studies have shown that the inclusion of equatorial chloride ligands has a notable effect on the results of uranyl electronic spectra calculations. When calculating the electronic spectrum of [UO_2_]^2+^ and [UO_2_Cl_4_]^2–^, Pierloot et al.,^24,48^ showed that the presence of the chloride ligands leads to a blueshift in the position of excited states and further changes the character of the luminescent state between the two systems. Gomes et al.,^49^ later showed that capturing the Cs_2_UO_2_Cl_4_ crystal environment more completely through incorporation of the Cs counterions had only a small improvement on predicting the relative position of excited states. A recent 4c-DR-TDDFT simulation of uranyl O K-edge XANES by Misael et al.,^50^ showed that relative peak positions can be brought into closer agreement with experiment by the inclusion of equatorial chloride ligands. However, this study also showed that the modeling of the counterions as an embedding potential did not significantly alter predictions. We have recently reported uranyl O K-edge simulations utilizing a free uranyl dication model ([UO_2_]^2+^),^42^ neglecting any potential influence of the equatorial ligands and surrounding cesium counterions on the predicted XANES spectrum. This study, in contrast, investigates the importance of including ligands and the broader chemical environment in XANES simulations and their impact when evaluating differences in covalency between the GS and CESs.

## Computational Method

**Figure 1:**
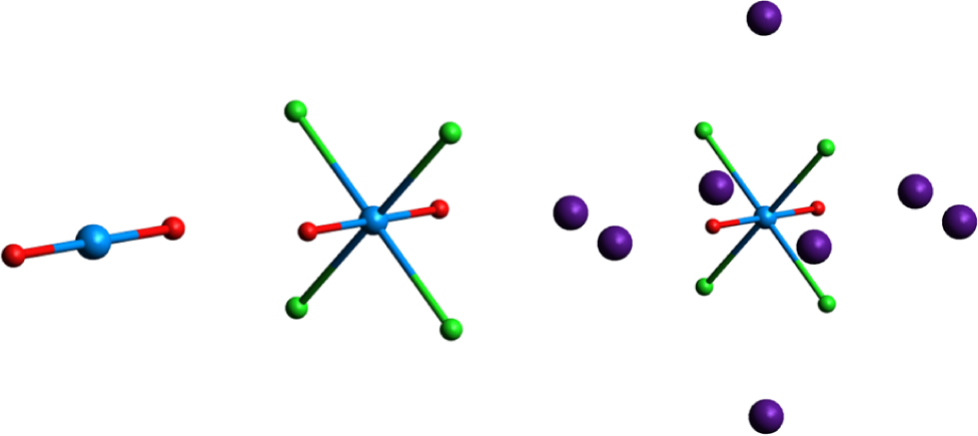
D_2h_ symmetry [UO_2_]^2+^, [UO_2_Cl_4_]^2–^, and Cs_2_UO_2_Cl_4_ structure models (from left to right) utilized in this study. The Cs_2_UO_2_Cl_4_ model makes use of 8 point-charges to represent Cs counterions (see ESI section 8).

Scalar-relativistic multiconfigurational XANES simulations were performed using version 21.02 of Openmolcas.^41,51−53^ Simulations were performed on three different idealized models (Figure 1) of the Cs_2_UO_2_Cl_4_ crystal utilized in the XAS experiments reported by Denning et al.^19,54^ Simulations utilized the highest abelian point-group of D_2h_ with [UO_2_]^2+^ and [UO_2_Cl_4_]^2–^ geometries constructed using experimentally reported bond lengths.^19,54^ For U–O and U–Cl bonds these correspond to 1.77 and 2.67 Å respectively. The local crystal environment of Cs_2_UO_2_Cl_4_ was modeled by using atomic centers with all-electron basis sets for the [UO_2_Cl_4_]^2–^ subunit, while the 8 cesium counterions of the local environment were modeled using dummy atoms with fractional +1/4 au point-charges to ensure charge neutrality. Point-charge positions and bond lengths relative to uranium reflect those of the XRD structure,^54^ with minimal adjustments made to retain overall D_2h_ symmetry (see ESI section 8). All-electron relativistic ANO-RCC TZVP-quality basis sets of Roos et al.,^55−57^ were employed, with higher angular momentum h-functions removed for uranium to allow for subsequent analysis. Basis sets were therefore of the form: U (9s8p6d4f2g), O (4s3p2d1f) and Cl (5s4p2d1f). Scalar-relativistic effects were modeled by use of the second order Douglas-Kroll-Hess Hamiltonian.^58−62^

**Figure 2:**
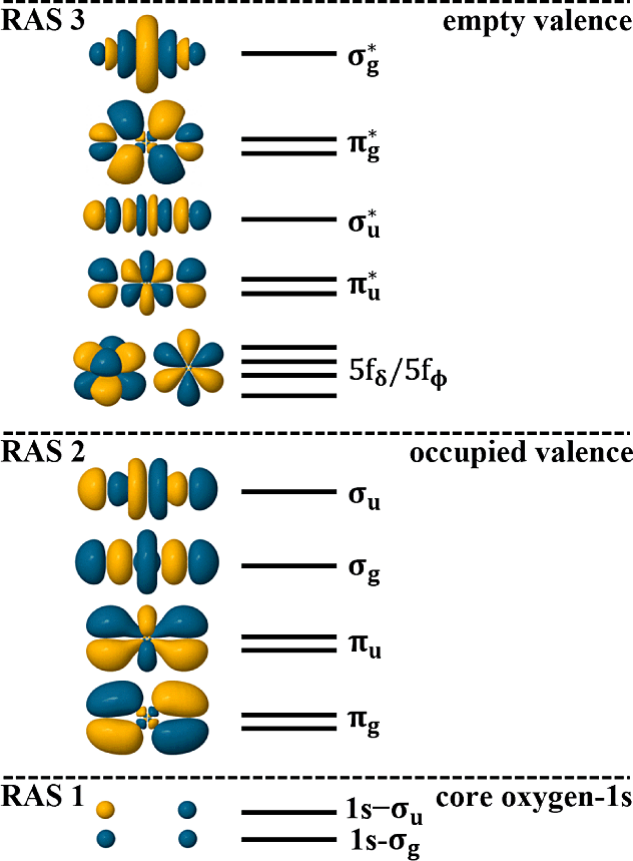
Active space used for RASSCF XANES simulations. Orbitals are the GS SONOs taken from the [UO_2_]^2+^ RAS(SD) simulations with isovalue of 0.03. Qualitative levels indicate the number of orbitals of each symmetry and do not reflect actual energy positions.

RASSCF theory was used to obtain the ground- and core-excited states.^39,41,63^ Unlike Complete Active-Space SCF (CASSCF) calculations which involve a full configuration interaction expansion on a select number of orbitals and electrons that form the active space,^38,40^ in RASSCF the active space is partitioned into three subspaces called RAS1, 2, and 3, as shown in Figure 2. Our general approach to simulating XANES is to place core-orbitals in RAS1, the occupied bonding orbitals in RAS2, and empty or partially filled valence orbitals in RAS3.^42^ This setup is comparable to approaches taken by others.^32,33^ for uranyl U M_4/5_-edge simulations. To generate the GS and CESs required for uranyl O K-edge simulation, the two linear combinations of oxygen 1s-orbitals (1s-σ_g_ and 1s-σ_u_) span RAS1, the six bonding orbitals (2 × π_g_, 2 × π_u_, σ_g_, σ_u_) span RAS2, and six antibonding orbitals () span RAS3. The four empty nonbonding 5f orbitals (2 × 5f_δ_ and 2 × 5f_ϕ_) were also included within RAS3. CESs were generated by allowing up to one or two electrons to occupy RAS3, and enforcing a single core-hole across RAS1: Using the RASSCF(*n,l,m*;*i,j,k*) notation adopted from Sauri et al.^64^ we hereafter refer to these two sets of simulations, RASSCF(16,1,1;2,6,10) and RASSCF(16,1,2;2,6,10), as RAS(S) and RAS(SD), respectively. The truncation of the CI expansion described above allows for a degree of control over the electron-correlation captured in simulations and manages computational cost. Due to the constraints on the active space in RAS(S) simulations, the only electrons populating RAS3 in the CESs will come directly from excitation of oxygen 1s orbitals. For this reason, RAS(S) simulations are utilized predominantly as an aid for peak assignments, see Figure S4 and Table S5 (ESI). RAS(S) calculations capture only limited static correlation and so higher-quality RAS(SD) data are presented within the main text, allowing comparison with our previously reported O K-edge and An M_4/5_-edge RAS(SD) results for [UO_2_]^2+^ and [NpO_2_]^2+^.^42^ Built-in supersymmetry designations of Openmolcas were used to restrict rotation of oxygen 1s linear combinations out of RAS1 during the SCF procedure, and the Laporte selection rule was imposed, meaning only CESs of ungerade (u) symmetry and a single GS of gerade (g) symmetry were required for state-interaction. The GS was obtained by removing the RAS1 core-hole constraint and taking the first root of a state-averaged singlet A_g_ calculation, with state-averaging required to stabilize nonbonding 5f orbitals within the active space. Both singlet and triplet spin-multiplicities are possible depending on the spin alignment of electrons in the CESs and were obtained by performing the appropriate state-averaged calculations across the odd-parity irreps (see ESI section 1). Our previous [UO_2_]^2+^ simulations made use of an energy cutoff to reduce the number of CESs supplied to RASSI,^42^ no such cutoff is used in this study for [UO_2_Cl_4_]^2–^ or Cs_2_UO_2_Cl_4_ RASSI calculations. State-specific second order RAS perturbation theory (RASPT2),^65,66^ with a default IPEA shift^67^ of 0.25 au and an imaginary shift^68^ of 0.5 au. was performed on RASSCF states to recover dynamic correlation and obtain quantitative state energies. The imaginary shift value offered a reasonable compromise between converging intruder free solutions without significantly affecting RASPT2 state energies and is comparable with values used in related studies.^14,31−33^ Ground and CESs were spin–orbit coupled *post hoc via* state-interaction of the scalar relativistic states with a mean-field spin–orbit operator, making use of atomic mean-field integrals (AMFI), in the Restricted Active Space State Interaction (RASSI) formulizm.^62,69^ Diagonal energies of the Hamiltonian matrix computed by RASSI were replaced by RASPT2 state energies. Spin orbit-coupled state energies and oscillator strengths between the GS and CESs were used to generate transition stick spectra, which were subsequently broadened by fitting Lorentzian functions to all transitions with shared full-width at half-maximum (fwhm) value of 0.8 eV. The choice of fwhm is considered arbitrary since the peak maxima are found to remain the same regardless of the value chosen, but 0.8 eV was found to offer good visual comparison with experiment as shown in figure S21. Only electric-dipole intense transitions are considered in this study, higher order multipole effects are not included. Spin–Orbit Natural Orbitals (SONOs),^32,70,71^ were utilized for peak assignment and subsequent covalency analysis as previously reported.^42^ Using SONOs to represent the electron density, Quantum Theory of Atoms in Molecules (QTAIM)^72,73^ analysis was performed using version 19.02.13 of AIMALL,^74^ and a variety of orbital composition analysis methods were performed using version 3.8 of Multiwfn.^75^

## Results and Discussion

### XANES Spectra

Core-excitations in uranyl O K-edge XANES occur between the RAS1 and RAS3 orbitals shown in Figure 2. Two different core-excitations can occur from oxygen 1s-σ_g_ into the valence  and  orbitals, and likewise from oxygen 1s-σ_u_ into valence  and  orbitals. Core-excitations into the nonbonding 5f-orbitals can also occur, but these transitions are expected to be of low intensity due to them formally containing no oxygen-2p component. The experimental XAS spectrum reported by Denning contains three main peaks at 531.4 eV, 534.1 and 536.5 eV, assigned to core-excitations from oxygen 1s orbitals into the , and  orbitals, respectively.^19^

**Figure 3:**
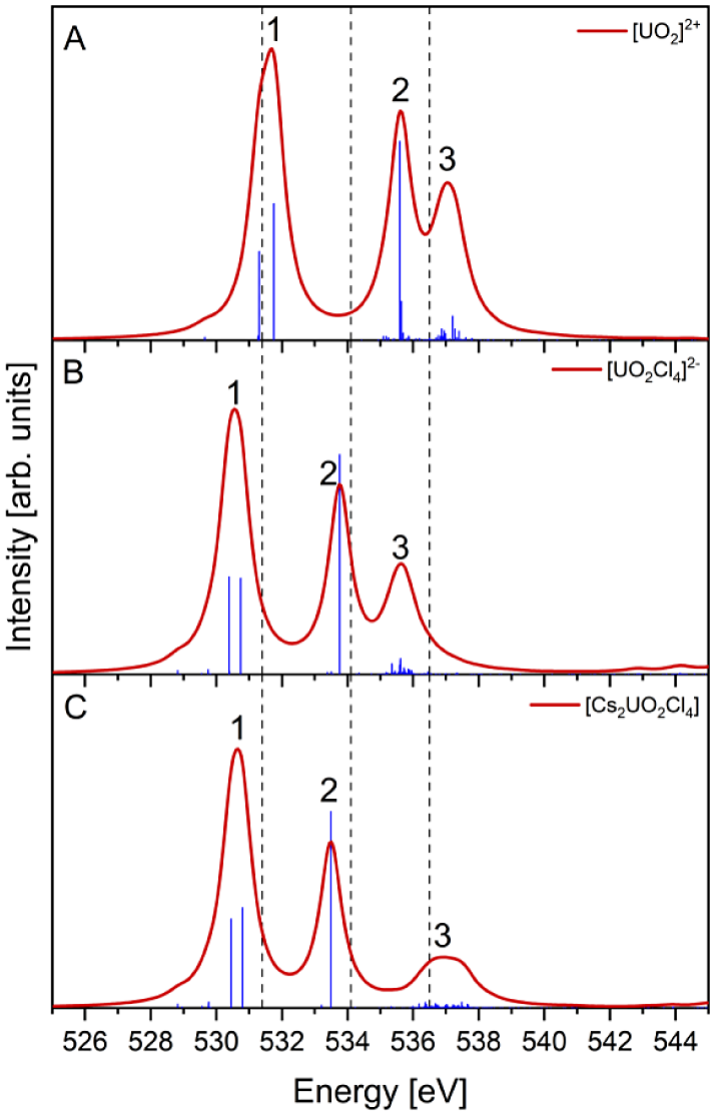
RAS(SD) O K-edge XANES simulations of (A) [UO_2_]^2+^,^42^ (B) [UO_2_Cl_4_]^2–^ and (C) Cs_2_UO_2_Cl_4_. Experimental positions are indicated by dashed vertical lines.^19^ [UO_2_]^2+^ data was reproduced from ref. 42. Peaks were generated using Lorentzian broadenings with a fwhm of 0.8 eV. No energy shift was applied to simulation data.

Two XAS spectra were obtained by Denning, one with the incident linearly polarized X-rays parallel to the O–U–O axis, and the other when perpendicular to this axis. The simulated XANES in this study do not consider X-ray polarization. All three uranyl models considered here resulted in simulated O K-edge spectra that predict the same three-peak structure within the 525–545 eV region consistent with experiment (Figure 3), and are assigned to the same core-excitations as those proposed by Denning. Two additional features observed in the experimental spectrum are also captured regardless of which uranyl model is used and are highlighted in Figures S4–7 (ESI). These features include a weak intensity shoulder on the lowest energy peak at ∼530 eV and a set of more intense features at higher energy, ∼550 eV. We restrict our focus to the three main peaks discussed above, labeled 1–3 in Figure 3.

Figure 3 and Table 1 report RAS(SD) calculations for all models and predict the uranyl O K-edge XANES peak positions with excellent accuracy, to within 1.5 eV of experiment. Of the three models simulated, the position of peak 1 is best predicted by the uranyl dication simulation, with a discrepancy of +0.3 eV. Peak 3 is also well predicted with a discrepancy of +0.6 eV. However, peak 2, predicted at 535.6 eV compared to the experimental position of 534.1 eV, a discrepancy of +1.5 eV, represents the largest predicted error of the three uranyl models. Equatorial chloride ligands included in the [UO_2_Cl_4_]^2–^ model have a stabilizing effect on the CESs relative to the GS, resulting in a redshift of all peaks with respect to both experiment and the [UO_2_]^2+^ simulation. Addition of equatorial chloride ligands moves peak 2 within 0.3 eV of the experimental peak position, but both peak 1 (Δ=-0.8 eV) and peak 3 (Δ=-0.9 eV) are then predicted with larger discrepancies than in the dicationic free uranyl model. The absolute magnitude of the discrepancies in peak predictions with respect to experiment can be made more consistent with the inclusion of cesium counterions modeled as point charges. By including the point charges in the Cs_2_UO_2_Cl_4_ model peaks 1 to 3 are predicted at 530.7 eV (Δ=-0.7 eV), 533.5 eV (Δ=-0.6 eV) and 537.0 eV (Δ=+0.5 eV), respectively.

**Table 1:** Simulated RAS(SD) Peak Energies Compared with Literature and Experimental Values^a^^b^

	peak 1	peak 2	Peak 3
Expt.^19^	531.4	534.1	536.5
[UO_2_]^2+^ Δ*S*CF^19,76^	531.8 (+0.4)	536.7 (+2.6)	537.3 (+0.8)
[UO_2_]^2+^ TDDFT^76^	510.0 (−21.4)	515.6 (−18.5)	516.9 (−19.6)
[UO_2_Cl_4_]^2–^ 4c-DR-TDDFT^50^	518.5 (−12.9)	521.4 (−12.7)	522.7 (−13.8)
[UO_2_]^2+^ RAS(SD)^42^	531.7 (+0.3)	535.6 (+1.5)	537.1 (+0.6)
[UO_2_Cl_4_]^2–^ RAS(SD)	530.6 (−0.8)	533.8 (−0.3)	535.6 (−0.9)
Cs_2_UO_2_Cl_4_ RAS(SD)	530.7 (−0.7)	533.5 (−0.6)	537.0 (+0.5)

a Values in brackets show discrepancy with respect to experiment.

b No energy shift was applied to the reported data.

Previous calculations of CESs using a Δ*S*CF approach for the free [UO_2_]^2+^ dication have been reported in the literature and compared with TDDFT calculations, the results of which are also presented in Table 1.^19,76^ Both RASSCF and Δ*S*CF approaches involve calculating the electronic structures of the GS and CESs separately, meaning the predicted state energies capture differences in orbital relaxations due to the differing electronic structures. In contrast, TDDFT simulations, which do not capture such relaxations, report peaks that are approximately 20 eV higher than experimental positions.^76^ Published 4c-DR-TDDFT calculations by Misael et al.^50^ show substantial improvement on peak predictions over the TDDFT reported in Table 1. These simulations included equatorial chloride ligands and the resulting discrepancy in peak positions is reduced to between 11–14 eV. Literature simulations therefore point to both the importance of including orbital relaxation in simulations, but also to the influence of the local environment on the prediction of peak positions. Both the Δ*S*CF and RAS(SD) simulations on the uranyl dication offer good predictions for peaks 1 and 3, but exhibit similar difficulty in predicting the absolute position of peak 2, with a discrepancy of +2.6 eV and +1.5 eV respectively. However, this large discrepancy in peak 2, corresponding to a core-excitation into the  orbital, is reduced to 0.3 eV in the [UO_2_Cl_4_]^2–^ RAS(SD) simulations. This substantial improvement indicates a particular sensitivity of peak 2 to the equatorial environment. This finding aligns with previous U M_4_-edge XANES studies that have also shown the position of the peak attributed to  to be highly sensitive to the molecular environment in which the uranyl unit is found.^77,78^

Table 2 reports the relative separation between the simulated peaks in Figure 3. Inspection of Table 2 reveals that the predicted separation between peaks 1 and 3 is in good agreement with the experimental separation (Δ= 5.1 eV) in both the [UO_2_]^2+^ (Δ = 5.4 eV) and [UO_2_Cl_4_]^2–^ (Δ = 5.0 eV) simulations. Discrepancies between experimental and predicted peak separations for the [UO_2_]^2+^ simulation result from predicting peak 2 at a higher energy relative to peaks 1 and 3, as compared with experiment. The discrepancies were improved with the inclusion of the equatorial chloride ligands in the [UO_2_Cl_4_]^2–^ simulation, which offers the closest agreement between the experimental and predicted peak separation of all the simulated models. The improved agreement resulting from the lowering in energy of peak 2 relative to both peaks 1 and 3 points once again to a greater sensitivity of the  orbital to the molecular environment compared to the other RAS3 orbitals. The inclusion of cesium counterions in the Cs_2_UO_2_Cl_4_ model is found to improve the relative separation between peaks 1 and 2 further compared to [UO_2_Cl_4_]^2–^, however, separation relative to peak 3 (Δ_32_ and Δ_31_) is impacted negatively due to peak 3 being pushed to higher excitation energy in the presence of the Cs point-charges. The shift in the position of peak 3 results from the spreading of transitions over a larger energy range, which broadens the peak and moves its maxima to higher energy.

**Table 2:** RAS(SD) O K-Edge Relative Peak Separations for [UO_2_]^2+^,^42^ [UO_2_Cl_4_]^2–^, and Cs_2_UO_2_Cl_4_^a^^b^

	Δ_21_	Δ_32_	Δ_31_
Expt.^19^	2.7	2.4	5.1
[UO_2_]^2+^	3.9 (+1.2)	1.5 (−0.9)	5.4 (+0.3)
[UO_2_Cl_4_]^2–^	3.2 (+0.5)	1.8 (−0.6)	5.0 (−0.1)
Cs_2_UO_2_Cl_4_	2.8 (+0.1)	3.5 (+1.1)	6.3 (+1.2)

a The separation between peaks A and B is given by Δ_AB_, and is calculated for peaks shown in Figure 3.

b Values in brackets show discrepancy with respect to experimental separations.^19^

### XANES Assignments

Simulated peaks were assigned by analyzing electron populations of the spin–orbit natural orbitals (SONOs) for a selection of intense core-excitations as shown in Tables S5–8 (ESI). Table 3 reports the populations of key CESs assigned to the most intense transitions in Figure 3. Peak characterization can be performed by examining the population of the RAS3 orbitals which are vacant in the GS but populated upon core-excitation. Due to the nature of the constraints on the RASSCF wave functions, all CESs will accommodate at least one electron in RAS3 directly from the oxygen 1s orbitals in RAS1 but can also be populated by electrons from the RAS2 bonding orbitals. In all O K-edge simulations, peak 1 can be attributed to two intense transitions into the  orbitals, with populations exceeding 0.96. Peak 2 is attributed to a single intense core-excitation into the  orbital populated above 0.68. Peak 3, however, is attributed to many low intensity transitions across a wide energy range. Assignment of peak 3 is more ambiguous due to the multiconfigurational nature of the states, with all RAS3 orbitals partially populated to various degrees. Despite this, the  orbitals for states associated with peak 3, have electron populations ranging from 0.20 to 0.26 in the three different models, which although low, is above the level at which these orbitals are populated across all other states. This character is emphasized in RAS(S) [UO_2_]^2+^ O K-edge simulations where peak 3 can be attributed to two intense transitions with dominant  occupancy of 0.90. Taking this into consideration, peak 3 is ultimately assigned to core-excitations into  for RAS(SD) simulations.

**Table 3:** RAS(SD) [UO_2_]^2+^,^42^ [UO_2_Cl_4_]^2–^ and Cs_2_UO_2_Cl_4_ O K-Edge SONO Populations of RAS3 Orbitals for CESs Associated with Key Transitions Responsible for Peaks in Figure 3^a^

model	peak	5f_δ/ϕ_				
[UO_2_]^2+^	1	0.04	0.99	0.08	0.16	0.00
	2	0.64	0.16	0.68	0.08	0.01
	3	1.02	0.54	0.05	0.26	0.00
[UO_2_Cl_4_]^2–^	1	0.02	1.00	0.09	0.17	0.00
	2	0.10	0.17	0.95	0.13	0.00
	3	0.97	0.69	0.04	0.20	0.00
Cs_2_UO_2_Cl_4_	1	0.06	0.96	0.06	0.18	0.00
	2	0.07	0.14	0.97	0.14	0.00
	3	1.21	0.42	0.04	0.22	0.00

a Tables S5–8 (ESI) provide populations for all orbitals in the active-space as well as for other CESs sampled.

In all uranyl simulations, multiconfigurational character of the states is found to increase with increasing excitation energy from peaks 1 to 3. This manifests as increasing partial occupation of RAS3 valence orbitals by either the excited 1s-electron or via partial depopulation of bonding orbitals. For example, the total RAS3 population in Table 3 for Cs_2_UO_2_Cl_4_ is 1.26, 1.32, and 1.89 electrons for the key CESs assigned to peaks 1 to 3 respectively. The excess electron population above 1.00 is accounted for by depletion of the bonding orbitals in RAS2, which are redistributed across RAS3. The 5f_δ_/5f_ϕ_ and  orbitals tend to be most significantly populated across the CESs regardless of the overall assignment and suggests that the partial occupation of these lower energy orbitals offers an energetically favorable route for optimizing the CESs in response to the core-hole and associated additional electron populating the valence space. Similar redistribution of electrons is found in the work of Autschbach and co-workers when using similar RAS methodologies,^14,31,32,35^ particularly for simulations involving the [U(C_7_H_7_)^2–^] system.^30^

Some additional features are also predicted by the simulations in all three models, including a shoulder on peak 1, which is also present at ∼530 eV in experiment and attributed to weak transitions into the nonbonding 5f-orbitals. Further features are predicted by simulation in the post 540 eV region, see Figures S4–7 (ESI), and are also identified in experiment. In the RAS(SD) simulations of [UO_2_]^2+^ and [UO_2_Cl_4_]^2–^, these post 540 eV features result from transitions involving CESs with substantial  orbital occupation. For Cs_2_UO_2_Cl_4_, the CESs responsible for similar features do not present significant  occupation in the states sampled, and instead electrons are distributed across all other RAS3 orbitals. Overall, the outlined assignment of the peaks agrees with those of Denning and others,^18,19,76^ and suggests an energetic ordering of the empty valence orbitals in uranyl as follows: .

### Intensity Profile

**Figure 4:**
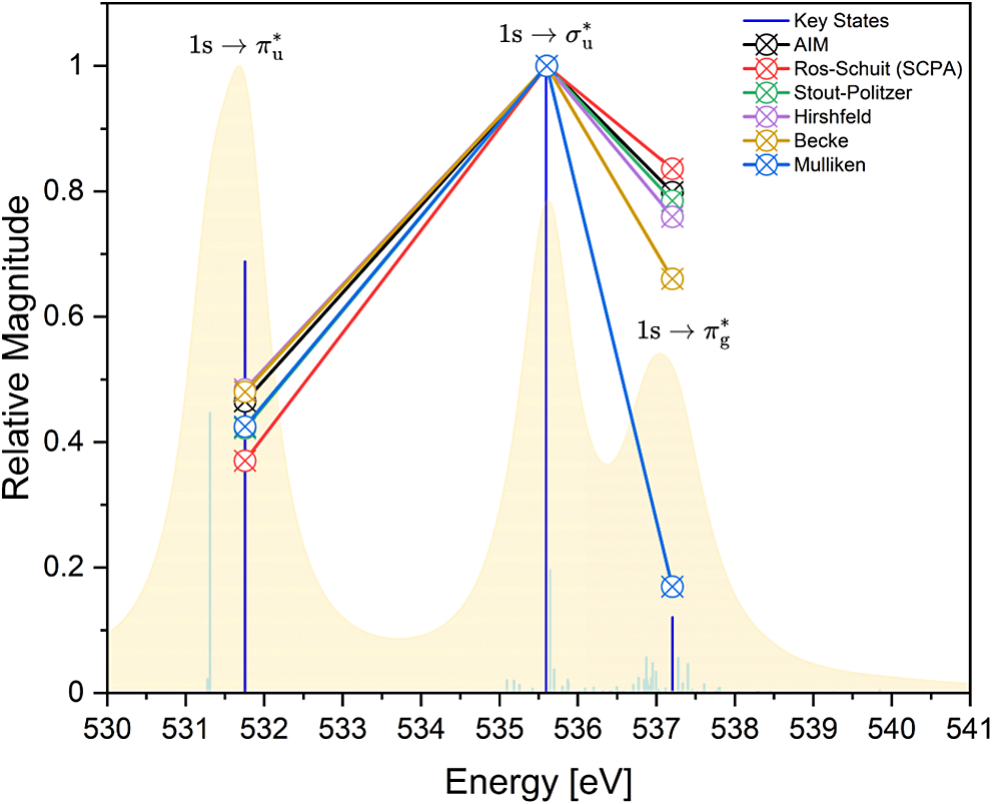
Relative trends in total oxygen contribution to the occupied antibonding orbital for representative [UO_2_]^2+^ RAS(SD) CESs assigned to peaks 1–3 in Table 3, using various composition methods.^75,79−86^ Trends are shown with respect to the normalized RAS(SD) XANES profile and transition stick spectra. [UO_2_]^2+^ spectrum data was reproduced and adapted from ref. 42.

All simulated spectra shown in Figure 3 present a similar three peak profile, with decreasing intensity from peaks 1 to 3. In all models, the intensity of peak 1 is attributed to two intense transitions and peak 2 is attributed to a single intense transition. The overall lower intensity profile of peak 3 in all models is attributed to a large number of relatively low intensity transitions spread across a wide energy range compared to peaks 1 and 2. Transitions attributed to peak 3 are found to span a greater energy range with the inclusion of equatorial chloride ligands and Cs point-charges, with both serving to progressively broaden and lower the intensity of the peak. Experimentally, the peak intensities of O K-edge spectra are generally interpreted as being driven by the degree of oxygen 2p-character in the antibonding orbitals and so it is informative to determine if such a relationship can be established theoretically. In Denning’s XAS study,^19^ two polarized absorption spectra were taken, one with the incident X-ray perpendicular to the O–U–O axis and the other parallel to this axis. Both the experimental absorption spectra present similar three peak profiles in the 525–545 eV range. However, the Cs_2_UO_2_Cl_4_ crystal was not perfectly aligned with the incident X-ray beam, resulting in a degree of polarization mixing. A consequence of this is that measured peak intensities, as highlighted by Denning,^19^ are only qualitative in nature, and as such we do not attempt to directly compare experimental intensities to simulated peak intensities obtained by Lorentzian broadening of transitions. Instead, we assess if there is a link between the magnitude of the oscillator strengths for key transitions (those assigned in Table 3) and the degree of oxygen character within an antibonding orbital associated with that transition. This analysis is performed for the simulated RAS(SD) [UO_2_]^2+^ spectrum. The oxygen contribution to the , and  antibonding orbitals for transitions assigned to peaks 1, 2 and 3, respectively, were obtained using a variety of orbital composition analysis methods.^75,79−86^ Orbital composition analysis was performed directly on SONOs or on the electron densities generated using SONOs for AIM analysis. The compositions for a given transition and orbital of interest were normalized with respect to the highest global O% across the transitions and orbitals considered, enabling relative trends to be compared. See Tables S16 and 17 in the ESI for details. To facilitate comparison with AIM, Becke, and Hirshfeld approaches in Figure 4, the breakdown of the oxygen contribution into the amount of p-character is not considered here using the Mulliken-like methods. Rather, it is assumed that the only atomic orbitals contributing to the antibonding orbitals of interest in uranyl come from U and O(2p) orbitals. This assumption is valid for the π*-orbitals, with Mulliken-like methods predicting oxygen contribution to be of p-character. In contrast, the σ*-orbital allows substantive sp-hybridization, however Tables S16 and 17 (ESI) show that p-character contributions in relation to the oscillator strengths lead to similar conclusions as discussed here. Figure 4 is generated by normalizing the magnitudes of individual transition oscillator strengths and peak intensities from the RAS(SD) [UO_2_]^2+^ spectrum with respect to the maximum values, allowing the trends in relative oxygen contributions to the antibonding orbitals to be compared to the relative strength of the transitions they are associated with. It was expected that the transitions with the largest oscillator strength would correspond to CESs with the greatest oxygen contributions to the antibonding orbitals. In this case, following the order of the highest to lowest oscillator strength, the largest oxygen character would be expected for the  transition, followed by  and then . All methods find the expected higher relative oxygen contribution in the  orbital compared to , aligning with the lower transition oscillator strength in the latter. Furthermore, all methods predict the expected lower oxygen contribution to the  orbitals in relation to the  orbitals. However, with the exception of Mulliken analysis, oxygen contributions are predicted to be relatively larger in the  orbitals compared to , despite the oscillator strength being much greater in magnitude for the transition associated with the latter. The same qualitative trends are also reported in the ESI for the lower-level RAS(S) simulation on [UO_2_]^2+^. With all other methods predicting a larger than expected oxygen contribution to the  orbitals for the  transition with respect to the low magnitude of the oscillator strength, the Mulliken oxygen composition for this transition should be viewed as anomalous.

In RASSCF calculations, orbitals can relax in order to better reflect the change in electronic structure between the GS and CESs. Notable changes in the electronic structures between the GS and CESs are shown to occur in the data presented in Tables 4–6 and are discussed in greater detail in the following section. Therefore, these data demonstrate that the oscillator strengths, while strongly influenced by the specific oxygen character in the antibonding orbitals of the CES, also reflect additional and more complex changes in the overall electronic structure between the GS and CESs. Consequently, the analysis of oxygen character solely in the  orbital is not adequate to capture these larger changes between the GS and the CES, and by extension the magnitude of the transition oscillator strength generated by the associated transition.

**Table 4:** Changes in Delocalisation Index Δδ(U, O) and Localisation Indexes Δλ(X) between the GS and CESs for Different Uranyl Models, Corresponding to Differences in Values Reported in Table S9 (ESI)^a^

model	property			
[UO_2_]^2+^	Δδ(U,O)	–0.55	–0.62	–0.68
	Δλ(U)	+0.71	+0.83	+0.99
	Δλ(O)	+0.20	+0.22	+0.21
[UO_2_Cl_4_]^2–^	Δδ(U,O)	–0.42	–0.43	–0.57
	Δλ(U)	+0.69	+0.68	+0.98
	Δλ(O)	+0.09	+0.10	+0.13
Cs_2_UO_2_Cl_4_	Δδ(U,O)	–0.42	–0.42	–0.55
	Δλ(U)	+0.69	+0.66	+1.00
	Δλ(O)	+0.09	+0.10	+0.11

a [UO_2_]^2+^ data taken from previously published simulations.^42^

### Covalency Analysis

The goal of this section is to quantify the extent to which O K-edge XANES is a reliable probe of GS uranyl covalency. For this to be the case, it is a requirement that the bonding orbitals in the CESs be sufficiently similar to those in the GS. We have previously reported that QTAIM analysis paired with AIM orbital composition determination allows changes in covalency to be quantified.^42^ Our prior work demonstrated that bonding orbitals in [UO_2_]^2+^ O K-edge CESs undergo notable relaxation, resulting in reduced uranium bonding contributions when compared to the GS. However, an outstanding question remains: Does the presence of chloride ligands and counterions mitigate the effects of this orbital relaxation? A similar combination of QTAIM and AIM composition analysis was employed to explore this question for the three uranyl models under consideration.

### QTAIM Analysis

QTAIM analysis was employed to investigate changes in covalency between the GS and CESs for all three uranyl models. An established measure of covalency is the magnitude of the electron density at the bond critical point, ρ_BCP_, reported in Tables S10–S12 (ESI) which measures charge accumulation in the bonding region through σ-interactions. In the GS, the ρ_BCP_ of [UO_2_]^2+^ is 0.33 au, indicative of substantial covalency.^11,44^ The addition of equatorial chloride ligands and Cs point-charges does not significantly alter this value for the U–O bond, retaining values of 0.31 and 0.32 au for [UO_2_Cl_4_]^2–^ and Cs_2_UO_2_Cl_4_ respectively. Equatorial ligands in uranyl typically bond with uranium through largely electrostatic interactions and the GS ρ_BCP_ values of 0.06 au for U–Cl bonds in [UO_2_Cl_4_]^2–^ and Cs_2_UO_2_Cl_4_ are consistent with this picture.^43,45,47,48^

In all three models, the U–O ρ_BCP_ values exhibit only minor decreases in the CESs, changing by no more than 0.06 atomic-units with respect to the GS values. Only limited variation is expected since bonding orbital populations are not depleted substantially during core-excitations and populated antibonding orbitals are expected to contribute only minimally. A complementary measure of covalency, the delocalization index, δ(X, Y), reported in Table S9 (ESI), quantifies the number of electrons shared between two atomic centers X and Y, and can, in the absence of bond polarization, be considered a measure of bond order. This metric can be large in the absence of charge accumulation in the bond, is influenced by all orbital interactions and is found to be more sensitive to changes in covalency in this instance. Combining the delocalization index with the localization index, λ(X), which quantifies the number of electrons localized on an atomic center X, enables useful insight into the distribution of electrons in the GS and CES. In the GS, δ(U,O) for [UO_2_]^2+^ is 1.85, which decreases to 1.53 (Δ=-0.32) in [UO_2_Cl_4_]^2–^. The GS localization indices also change between [UO_2_]^2+^ and [UO_2_Cl_4_]^2–^, from 7.70 to 8.03 (Δ=+0.33) on oxygen and 86.85 to 86.37 (Δ=-0.48) on uranium, respectively. These changes collectively indicate a reduction in the number of electrons shared in the U–O bond and an enhancement of U–O ionic character upon the addition of electron withdrawing chloride ligands. Similar changes in U–O bonding interactions are also reflected when comparing the [UO_2_]^2+^ and Cs_2_UO_2_Cl_4_ ground-states, with the presence of cesium counterions having only a minor impact on QTAIM metrics in comparison to [UO_2_Cl_4_]^2–^.

The δ(U,Cl) and λ(Cl) values for the U–Cl bonds remain largely unchanged between the GS and the CESs, with values of approximately 0.40 and 17.6 respectively. These changes are reported in Tables S11 and 12 (ESI). Table 4 reports changes in U–O QTAIM metrics between the GS and CESs for all three models. δ(U,O) values were expected to decrease in the CESs due to the occupation of antibonding orbitals which acts to lower the uranyl bond order. This expectation was confirmed by decreases in the δ-values in Table 4. The electrons no longer shared in U–O bonding interactions in the CESs instead localize onto both the uranium and oxygen centers as indicated by increased localization indexes. For example, the  excitation in Cs_2_UO_2_Cl_4_ leads to a decrease in δ(U,O) from 1.52 to 1.10 (Δδ=-0.42) and consequent increases in Δλ(U) and Δλ(O) of +0.66 and +0.10 respectively. The degree to which δ(U,O) values decrease between the GS and CESs for a given model correlates with the increasing multiconfigurational nature of CESs from peaks 1 to 3. For example, in Cs_2_UO_2_Cl_4_, the δ(U,O) of 1.11 is larger in the 1s  CES, reducing to 0.97 for the 1s CES, where greater multiconfigurational character is manifested. The reduced δ(U,O) values in CESs with pronounced multiconfigurational character results from greater redistribution of electrons between RAS2 and RAS3 orbitals for these states. This electron redistribution depletes bonding orbitals while populating nonbonding and antibonding orbitals, collectively reducing the U–O bond order. The enhanced electron redistribution into the RAS3 orbitals, which all have predominantly uranium character, also explains why the largest Δλ(U) values are found for the 1s CESs.

Comparing δ(U,O) values in Table S9 (ESI) indicates U–O bonding within the CESs themselves is influenced by the molecular environment. Lower δ(U,O) values are found in the CESs of the chloride models compared to those in [UO_2_]^2+^: in particular, values range from 0.96 to 1.10 in [UO_2_Cl_4_]^2–^, while in [UO_2_]^2+^, they range from 1.17 to 1.30. The presence of the chloride ligands also impacts the degree to which reduction in δ(U,O) values occurs between the GS and CESs. Notably, the largest decrease in δ(U,O) between the GS and CESs occurs for [UO_2_]^2+^, with Δδ(U,O) values ranging from 0.55–0.68, and concurrent increase to both Δλ(U) and Δλ(O) spanning 0.71–0.99 and 0.20–0.22, respectively. In [UO_2_Cl_4_]^2–^, decreases in Δδ(U,O) are less pronounced, ranging from 0.42–0.57, and Δλ(O) increases are approximately halved, spanning 0.09–0.13. Increases in Δλ(U) are comparable to those in [UO_2_]^2+^, ranging from 0.69–0.98. The smaller Δδ(U,O) values in [UO_2_Cl_4_]^2–^ as compared to [UO_2_]^2+^ demonstrates a smaller reduction in U–O electron sharing between the GS and the CES in the former. This behavior can be attributed to more electron-rich oxygen centers in the [UO_2_Cl_4_]^2–^ GS, displaying localization indexes approximately 0.30 greater on each center compared to those in [UO_2_]^2+^. During excitation, electrons shared in U–O bonding interactions are liberated and localize onto each atomic center. Given the electron-rich nature of the oxygen centers in the [UO_2_Cl_4_]^2–^ GS, a greater energetic cost can be associated with further increasing electron localization on these centers in comparison to [UO_2_]^2+^. Consequently, the U–O covalency, as quantified in terms of electron sharing between the GS and CESs, differs to a lesser degree. This reasoning extends to Cs_2_UO_2_Cl_4_, whose QTAIM metrics mirror those of [UO_2_Cl_4_]^2–^.

### Orbital Composition Analysis

The extent to which the GS and CES electronic structures differ can also be examined through orbital composition analysis of the bonding SONOs. The RASSCF approach utilized in this study optimizes the GS and CESs via separate simulations, meaning changes in the bonding orbitals due to the presence of the core-hole and excited electron are captured. As shown in Figure 4, a variety of composition methods can be utilized and with the exception of Mulliken analysis, the methods presented all give comparable composition trends for orbitals in the CESs. The Atoms In Molecules (AIM) approach was chosen to generate compositions in line with our previous study.^42^ Orbital composition using an AIM approach partitions the molecular space to evaluate the total contribution of uranium and oxygen atomic basins to the SONOs (Tables 5 and 6). Analysis is presented for the bonding SONOs as opposed to the antibonding SONOs, due to the former remaining strongly occupied in the CESs, allowing more appropriate comparison with GS orbitals. The validity of O K-edge XANES as a probe of GS uranyl bonding can therefore be assessed, and the influence of the molecular environment on this assessment can be determined.

**Table 5:** Uranium Percentage AIM Orbital Compositions for Bonding SONOs for the Ground- and Key Core Excited-States Responsible for Peaks in Figure 3^a^^b^^c^^d^

		[UO_2_]^2+^		[UO_2_Cl_4_]^2–^		Cs_2_UO_2_Cl_4_	
Excitation	SONO	GS% → CES%	Δ	GS% → CES%	Δ	GS% → CES%	Δ
	π_u_	25% → 15%	–10%	20% → 13%	–7%	19% → 13%	–7%
	σ_u_	53% → 46%	–7%	48% → 41%	–7%	48% → 41%	–7%
	π_g_	17% → 9%	–8%	12% → 7%	–5%	12% → 8%	–4%

a Table reports the values from ground- to core excited-states, GS% → CES%, and the overall change in brackets.

b Analysis is from RAS(SD) electron densities.

c Full Table of compositions available in Tables S13–15 (ESI).

d [UO_2_]^2+^ data taken from previously published simulations.^42^

**Table 6:** Total Oxygen Percentage AIM Orbital Compositions for Bonding SONOs for the Ground- and Key Core Excited-States Responsible for Peaks in Figure 3^a^^b^^c^^d^

		[UO_2_]^2+^		[UO_2_Cl_4_]^2–^		Cs_2_UO_2_Cl_4_	
Excitation	SONO	GS% → CES%	Δ	GS% → CES%	Δ	GS% → CES%	Δ
	π_u_	75% → 85%	+10%	79% → 86%	+7%	80% → 84%	+4%
	σ_u_	47% → 54%	+7%	50% → 46%	–4%	50% → 44%	–6%
	π_g_	83% → 91%	+8%	84% → 91%	+7%	86% → 86%	0%

a Table reports the values from ground- to core excited-states, GS% → CES%, and the overall change in brackets.

b Analysis is from RAS(SD) electron densities.

c Full Table of compositions available in Tables S13–15 (ESI).

d [UO_2_]^2+^ data taken from previously published simulations.^42^

In the GS, bonding SONO compositions reported in Table 5 reveal lower U% contributions in chloride models compared to those in [UO_2_]^2+^. Consistent with QTAIM findings from the previous section, the lower U% contributions in chloride models also indicate lower U–O covalent mixing in comparison with [UO_2_]^2+^, as values deviate to a greater extent from the maximal 50:50 mixing. In each model, the GS %-contributions in Tables 5 and 6 reveal a trend in the bonding orbitals themselves, with decreasing covalency from σ_u_ through π_u_ to π_g_ for each model, with decreasing U% and increasing O% contributions leading to greater deviation from maximal covalent mixing. While the σ_u_ bonding orbital reflects strong covalent mixing across the GSs of all three models (U:O mixings of 53%:47% and 48%:50% for [UO_2_]^2+^ and Cs_2_UO_2_Cl_4_, respectively), π-bonding orbitals display increased ionic character when chloride ligands are present. For instance, there is a notable shift from 17%:83% U:O mixing for π_g_ in [UO_2_]^2+^, to 12%:84% in [UO_2_Cl_4_]^2–^ and 12%:86% in Cs_2_UO_2_Cl_4_ (note that the omitted Cl% contributions, reported in Tables S14 and 15, are required to recover compositions of 100% in chloride systems).

In our prior research simulating O K-edge of [UO_2_]^2+^,^42^ comparative analysis of orbitals between the GS and CESs demonstrated 7–10% lower U%-contributions to bonding SONOs in the latter. Table 5 recapitulates these findings, supplemented by oxygen basin contributions detailed in Table 6, highlighting the expected 7–10% increase in O% to CES bonding SONOs. A similar reduction in U% between GS and CESs is observed in the chloride models, with a consistent 4–7% decrease in U% contributions to bonding orbitals. Across all three models, the reduction in U% contributions are a consistent 7% for the *σ*_*u*_ orbital. However, for the chloride models the reduction in U% contributions to π-bonding orbitals is found to be less pronounced in comparison to [UO_2_]^2+^. This is particularly evident for the *π*_*g*_ orbitals, with an 8% reduction in U% contribution for [UO_2_]^2+^ and a 4–5% reduction for the chloride models. In the latter, GS π-bonding orbitals are more ionic in nature, with greater charge density residing on O centers compared to those of [UO_2_]^2+^. Therefore, upon core-excitation, there is a higher energetic cost associated with redistributing charge density in π-orbitals toward the oxygen centers in chloride models, accounting for the smaller decreases in U%. In contrast, the GS *σ*_*u*_ orbitals have similar covalent mixings across all three models, and therefore redistributing charge density onto oxygen centers in the  CESs will incur a similar energetic cost in all three models.

Two effects are thought to account for the reported reduction in U% contributions to bonding orbitals in the CESs: First, localization of charge density in the CES onto uranium is driven by the occupation of nonbonding 5f orbitals and antibonding orbitals with predominantly metal character, which repels density in the bonding orbitals away from the U center. Second, the increased effective nuclear charge of oxygen centers due to the core-hole attracts charge density in the bonding orbitals toward these centers. In free uranyl, these effects combine to account for both the reduced U% and increased O%. Here, the oxygen ligands are the sole atomic centers available to facilitate charge redistribution away from uranium in the CES bonding orbitals. The inclusion of chloride ligands, however, introduces an additional route for redistributing charge density away from uranium after core-excitation, and consequently, their presence is found to influence bonding orbital compositions in the CESs. This is most prominent for the σ_u_ orbital resulting from the  excitation for all three models. In [UO_2_]^2+^, the 7% reduction in uranium contribution to the *σ*_*u*_ orbital is accounted for by a necessary equal increase in O%. In [UO_2_Cl_4_]^2–^ and Cs_2_UO_2_Cl_4_, the 7% uranium reduction coincides with notable 4% and 6% reductions in oxygen contributions to the *σ*_*u*_ orbitals, respectively. These reductions in both U and O %-contributions are instead compensated by an approximate 11% and 12% increase in Cl%, respectively. Another notable example is the  excitation in Cs_2_UO_2_Cl_4_, with a 4% uranium decrease in the CES *π*_*g*_ orbital, which is compensated by a 4% increase in Cl%, with no significant change in O%. For all other excitations considered in Tables 5 and 6, reduction in U% contributions to bonding orbitals in chloride models are accounted for by some degree of increase in O%, with outstanding compositional changes accounted for by variations in Cl%.

## Conclusions

A RASSCF approach was employed to simulate uranyl O K-edge XANES across models that progressively improve the representation of the local crystal environment. Simulations successfully replicate the three peak-profile from XAS data of Denning et al.,^19^ and confirm the same peak assignments. Oxygen K-edge simulations employing the [UO_2_]^2+^ model yielded a spectrum with peak positions predicted within a 1.5 eV margin of experiment. Notably, the incorporation of equatorial ligands in the [UO_2_Cl_4_]^2–^ model refined peak predictions to within 1 eV and offered peak separations best aligning with experiment. Introducing cesium counterions in the Cs_2_UO_2_Cl_4_ model did not generally improve predictions. Instead, peak 3 was disproportionately impacted, with transitions spread across a large energy range leading to a broad peak that is pushed to higher excitation energy, and adversely affecting relative peak separations. Simulations highlight that O K-edge peaks can comprise of multiple transitions, and while single transitions can sometimes be identified as dominant (e.g., peak 2 in Figure 3), the states themselves exhibit significant multiconfigurational character. The degree to which the magnitude of the oscillator strength can be linked to a greater amount of O character in the antibonding orbital associated with a given transition, was examined using a variety of composition methods. Results show that the expected trend breaks down for the highest energy peak, with analysis methods identifying greater oxygen character in  orbitals compared to , despite the oscillator strength being greater for excitations into the latter. The magnitude of the oscillator strength is therefore not solely reflected in the oxygen 2p-character of the specific antibonding orbital associated with a transition but is also dependent on more complex changes in the overall electronic structure between the GS and CES.

The degree to which the covalency differs between the GS and CES was investigated for the three different models. The inclusion of cesium point charges in Cs_2_UO_2_Cl_4_ was found to have negligible impact on either the GS or CES QTAIM metrics compared to [UO_2_Cl_4_]^2–^, consistent with previous findings.^46^ In all three models, a reduction in δ(U,O) values and hence the U–O covalency, was found from the GS to the CES. The largest reductions in uranyl covalency across the models was found for the highly multiconfigurational  CESs, which present more significant electron redistribution from bonding to antibonding orbitals, and therefore a more substantial reduction in the uranyl bond order for these states. In contrast to [UO_2_]^2+^, U–O covalency between the GS and CES is found to decrease less substantially in chloride-containing models. The smaller reduction in U–O covalency is attributed to the more electron-rich oxygen centers in the GSs of these models, increasing the energetic cost of distributing electrons from shared U–O interactions onto oxygen centers in the CES when compared to [UO_2_]^2+^. Overall, QTAIM results indicate that GS covalency is better reflected in the CESs of the chloride-containing models as opposed to free [UO_2_]^2+^.

The extent to which CES bonding orbitals differ from those of the GS was investigated to determine the degree to which O K-edge XANES is a valid probe of GS orbital mixing. Previous research on [UO_2_]^2+^ demonstrated a 7–10% lower U-contribution to bonding orbitals in the CES compared to the GS.^42^ A similar finding is also reported here for both [UO_2_Cl_4_]^2–^ and Cs_2_UO_2_Cl_4_ models, with a 4–7% lower U-contribution to the bonding orbitals in the CESs. In [UO_2_]^2+^, reductions in U% between the GS and CESs necessitate commensurate increases in O%, while in the chloride-containing models, equatorial ligands offer an alternative route for the redistribution of charge density and are found to play a direct role in compositional changes. This is particularly evident for the CESs corresponding to the  excitation, where a reduction in both U% and O% contributions is accounted for by relatively large increases in Cl%. Incorporating equatorial ligands to enhance the representation of the crystal environment results in CES bonding orbitals that more closely align with GS compositions, and the further inclusion of Cs point-charges leads to even closer alignment. The smallest change in U% contribution between the GS and a CES is found in Cs_2_UO_2_Cl_4_, as well as overall CES oxygen contributions that better align with those of the GS. This underscores the importance of considering the crystal environment in theoretical calculations, especially when using such methods to assess the validity of XANES as a ground-state probe. However, our composition analysis ultimately reveals that probing O K-edge CESs does still lead to an underestimation of U% contributions to GS bonding orbitals. Further to this, analysis shows that the degree to which oxygen contributions are likely to be under- or overestimated will vary depending on the peak under investigation, and the model utilized.

## Supporting Information Available

Further computational details on the number of RASSCF states calculated. Additional results: Energetic data, details on peak assignments, further QTAIM and orbital composition data. Theoretical structure XYZ tables (PDF)
