# Supplementary material for: Quantifying Covalency and Environmental Effects in RASSCF-Simulated O K-Edge XANES of Uranyl
Source: Inorg Chem. 2024 Aug 2;63(32):15115–26. doi: 10.1021/acs.inorgchem.4c02144 (PMC11323269; doi:10.1021/acs.inorgchem.4c02144)
Supplement: Supplementary file 1 — ic4c02144_si_001.pdf [file ic4c02144_si_001.pdf]

Supporting Information for

Quantifying covalency and environmental effects in RASSCF-simulated O K-edge XANES of uranyl

Kurtis Stanistreet-Welsh[a] and Andrew Kerridge*[a]

[a] Department of Chemistry, Lancaster University, Lancaster LA1 4YB, UK

* a.kerridge@lancaster.ac.uk

Table of Contents:

1. Number of RASSCF and RASSI States 3

Table S1: Number of state-average RASSCF roots and states used in subsequent RASSI calculations for RAS(S) O K-edge XANES simulations for $[UO_2]^{2+}$ 3

Table S2: Number of state-average RASSCF roots and states used in subsequent RASSI calculations for RAS(SD) O K-edge XANES simulations for $[UO_2]^{2+}$ 3

Table S3: Number of state-average RASSCF roots and states used in subsequent RASSI calculations for RAS(SD) O K-edge XANES simulations for $[UO_2Cl_4]^{2-}$ and $Cs_2UO_2Cl_4$ 4

2. Energetic Data 4

Table S4: Table of peak energies from experiment, other studies and this work, which includes RAS(S) and RAS(SD) simulations for $[UO_2]^{2+}$, $[UO_2Cl_4]^{2-}$ and $Cs_2UO_2Cl_4$ 4

Figure S1: Shifted simulated RAS(SD) O K-edge peak measurments tabulated in table S4 5

Figure S2: Simulated O K-edge XANES peak measurments tabulated in table S4 5

Figure S3: Alternative shifted version of figure 3 in main manuscript 6

3. Additional Assignment Details/Plots 6

Figure S4: RAS(S) O K-edge XANES peak assignments for $[UO_2]^{2+}$ 6

Table S5: RAS(S) $[UO_2]^{2+}$ peak assignments 7

Figure S5: RAS(SD) O K-edge XANES peak assignments for $[UO_2]^{2+}$ 7

Table S6: RAS(SD) $[UO_2]^{2+}$ peak assignments 8

Figure S6: RAS(SD) O K-edge XANES peak assignments for $[UO_2Cl_4]^{2-}$ 9

Table S7: RAS(SD) $[UO_2Cl_4]^{2-}$ peak assignments 9-10

Figure S7: RAS(SD) O K-edge XANES peak assignments for $Cs_2UO_2Cl_4$ 10

Table S8: RAS(SD) $Cs_2UO_2Cl_4$ peak assignments for figure S6 11

4. Spin-Free Uranyl O K-edge XANES 12

Figure S8: Spin-Free RAS(SD) O K-edge XANES for $[UO_2]^{2+}$ showing peaks are generated by transitions belonging to B1u or B2u/B3u transition symmetries 12

Figure S9: Spin-Free RAS(SD) O K-edge XANES for $[UO_2]^{2+}$, $[UO_2Cl_4]^{2-}$ and $Cs_2UO_2Cl_4$ 12

Figure S10: Spin-Free RAS(SD) O K-edge XANES spectra for (a-b) $[UO_2]^{2+}$, (c-d) [UO2Cl4]2- and (e-f) $Cs_2UO_2Cl_4$ 13

5. QTAIM Metrics 14

Table S9: Summary of GS and CES QTAIM metrics for the three different uranyl models 14

Table S10: QTAIM analysis data for $[UO_2]^{2+}$ RAS(SD) states 14

Table S11: QTAIM analysis data for $[UO_2Cl_4]^{2-}$ RAS(SD) states 15

Table S12: QTAIM analysis data for $Cs_2UO_2Cl_4$ RAS(SD) states 16

6. Orbital Composition Analysis 17

Table S13: Atoms in molecules (AIM) percentage composition analysis for $[UO_2]^{2+}$ SONOs in RAS(SD) states 17

Table S14: Atoms in molecules (AIM) percentage composition analysis for $[UO_2Cl_4]^{2-}$ SONOs in RAS(SD) states 18-19

Table S15: Atoms in molecules (AIM) percentage composition analysis for $Cs_2UO_2Cl_4$ SONOs in RAS(SD) states 20

7. Oscillator Strengths versus Anti-Bonding Orbital Oxygen character 21

Table S16: Total oxygen percentage in the anti-bonding orbitals assigned to key core-excitations for $[UO_2]^{2+}$ RAS(SD) simulation using various methods....................................21

Table S17: Oxygen compositions from table S16 normalised and used to plot main text figure 4....................................21

Figure S11: Plot showing total oxygen percentage to anti-bonding orbitals for key transitions from the RAS(SD) $[UO_2]^{2+}$ simulation..........21

Figure S12: Plot showing the (a) relative normalised and (b) total oxygen percentage to anti-bonding orbitals for key transitions from the RAS(S) $[UO_2]^{2+}$ simulation....................................22

8. Structure Coordinates 22

Figure S13: U-O (1.77Å) and U-Cl (2.67Å) bond lengths in the $[UO_2Cl_4]^{2-}$ structure model....................................22

Figure S14: Bond lengths (Å) for the $Cs_2UO_2Cl_4$ structure model 23

Figure S15: Uranyl super cell utilized to inform $Cs_2UO_2Cl_4$ model 23

Table S18: $[UO_2]^{2+}$ D_{2h} X,Y,Z atomic coordinates....................................23

Table S19: $[UO_2Cl_4]^{2-}$ D_{2h} X,Y,Z atomic coordinates 23

Table S20: $Cs_2UO_2Cl_4$ D_{2h} X,Y,Z atomic coordinates 24

Figure S16: $Cs_2UO_2Cl_4$ structure showing atomic labels utilized in table S18....................................24

Table S21: Table of structure parameters for the experimental $Cs_2UO_2Cl_4$ system and the D_{2h} structure 24

9. Peak Broadenings....................................25

Figure S17: Lorentzian broadening of $[UO_2]^{2+}$ RAS(SD) transitions 25

10. References 25

1. Computational Details

1.1 Number of RASSCF and RASSI States:

The number of states for $[UO_2]^{2+}$ simulations are from a previous study. [1]

Table S1: Number of state-average RASSCF roots and states used in subsequent RASSI calculations for RAS(S) O K-edge XANES simulations for $[UO_2]^{2+}$.[1] GS = Ground-State, Core-ES = Core-Excited State.

	AG	AU	B1U	B2U	B3U
**GS Singlet**					
State-Average Performed:	3				
Used in RASSI:	1				
**Core-ES Singlets**					
State-Average Performed:		1	3	3	3
Total Possible Core-ESs:		1	3	3	3
Used in RASSI:		1	3	3	3
**Core-ES Triplets**					
State-Average Performed:		1	3	3	3
Total Possible Core-ESs:		1	3	3	3
Used in RASSI:		1	3	3	3

Table S2: Number of state-average RASSCF roots and states used in subsequent RASSI calculations for RAS(SD) O K-edge XANES simulations for $[UO_2]^{2+}$.[1] GS = Ground-State, Core-ES = Core-Excited State.

	AG	AU	B1U	B2U	B3U
**GS Singlet**					
State-Average Performed:	5				
Used in RASSI:	1				
**Core-ES Singlets**					
State-Average Performed:		145	155	155	155
Total Possible Core-ESs:		145	155	155	155
Used in RASSI:		62	63	68	68
**Core-ES Triplets**					
State-Average Performed:		217	221	221	221
Total Possible Core-ESs:		217	221	221	221
Used in RASSI:		93	90	95	95

Table S3: Number of state-average RASSCF roots and states used in subsequent RASSI calculations for RAS(SD) O K-edge XANES simulations for $[UO_2Cl_4]^{2-}$ and $Cs_2UO_2Cl_4$. GS = Ground-State, Core-ES = Core-Excited State.

	AG	AU	B1U	B2U	B3U
**GS Singlet**					
State-Average Performed:	5				
Used in RASSI:	1				
**Core-ES Singlets**					
State-Average Performed:		145	155	155	155
Total Possible Core-ESs:		145	155	155	155
Used in RASSI:		145	155	155	155
**Core-ES Triplets**					
State-Average Performed:		217	221	221	221
Total Possible Core-ESs:		217	221	221	221
Used in RASSI:		217	221	221	221

2. Energetic Data:

Table S4: Table of peak energies from experiment,[2] other studies[1,3] and this work, which includes RAS(S) and RAS(SD) simulations for $[UO_2]^{2+}$, $[UO_2Cl_4]^{2-}$ and $Cs_2UO_2Cl_4$.

	**Shoulder**	**Peak 1**	**Peak 2**	**Peak 3**	**Peak 4**
Experiment [2]	530.0	531.4	534.1	536.5	551.0
ΔSCF(BP86) [3]		531.8 (+0.4)	536.7 (+2.6)	537.3 (+0.8)	
TD-DFT(BP86) QZ4P [3]		510.0 (-21.4)	515.6 (-18.5)	516.9 (-19.6)	
$[UO_2]^{2+}$ RAS(S) [1]	530.2 (+0.2)	532.1 (+0.7)	536.1 (+2.0)	538.6 (+2.1)	
$[UO_2]^{2+}$ RAS(S) - 0.60eV	529.7 (-0.3)	531.4 (0.0)	535.5 (+1.4)	538.0 (+1.5)	
$[UO_2]^{2+}$ RAS(SD) [1]	529.7 (-0.3)	531.7 (+0.3)	535.6 (+1.5)	537.1 (+0.6)	551.7 (+0.7)
$[UO_2]^{2+}$ RAS(SD) - 0.30eV	529.4 (-0.6)	531.4 (0.0)	535.3 (+1.2)	536.8 (+0.3)	551.4 (+0.4)
$[UO_2Cl_4]^{2-}$ RAS(SD)	528.8 (-1.2)	530.6 (-0.8)	533.8 (-0.3)	535.6 (-0.9)	
$[UO_2Cl_4]^{2-}$ RAS(SD) + 0.85eV	529.7 (-0.3)	531.4 (0.0)	534.6 (+0.5)	536.5 (0.0)	
$Cs_2UO_2Cl_4$ RAS(SD)	528.9 (-1.1)	530.7 (-0.7)	533.5 (-0.6)	537.0 (+0.5)	
$Cs_2UO_2Cl_4$ RAS(SD) + 0.75eV	529.6 (-0.4)	531.4 (0.0)	534.3 (+0.2)	537.8 (+1.3)	

.

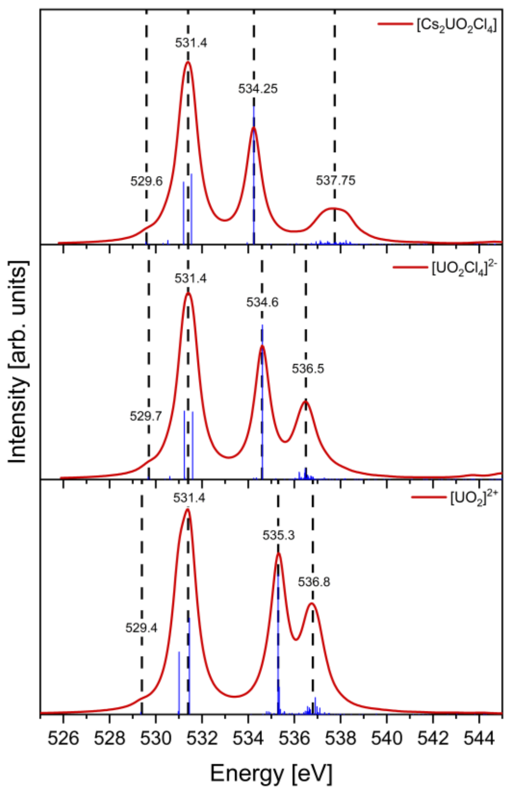

Figure S1: Shifted simulated RAS(SD) O K-edge peak measurments. $[UO_2]^{2+}$ data taken from simulations reported in ref 1. Shifted to align the first simulated peak with experiment to give a peak position of 531.4eV. Peak positions are then tabulated in table S4.

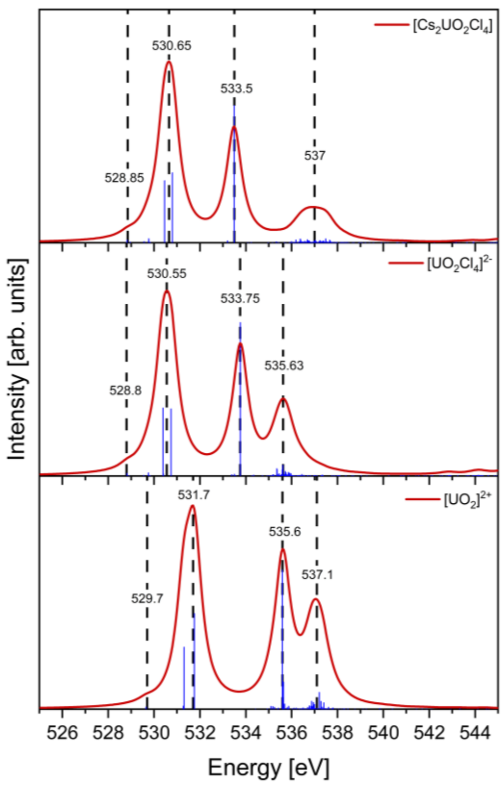

Figure S2: Simulated O K-edge XANES peak measurments tabulated in table S4. $[UO_2]^{2+}$ data taken from simulations reported in ref 1.

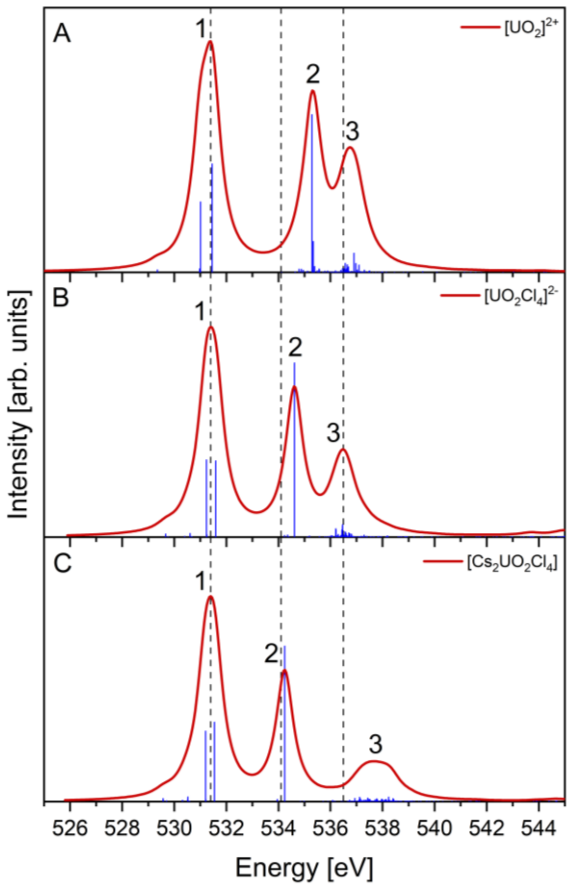

Figure S3: RAS(SD) O K-edge XANES simulations of (A) $[UO_2]^{2+}$,[1] (B) $[UO_2Cl_4]^{2-}$ and (C) $Cs_2UO_2Cl_4$. Experimental positions are indicated by dashed vertical lines.[2] Peaks were generated using Lorentzian broadenings with a FWHM of 0.8 eV. An energy shift was applied to simulation data to align the first simulated peak with experiment.

3. Additional Assignment Details/Plots:

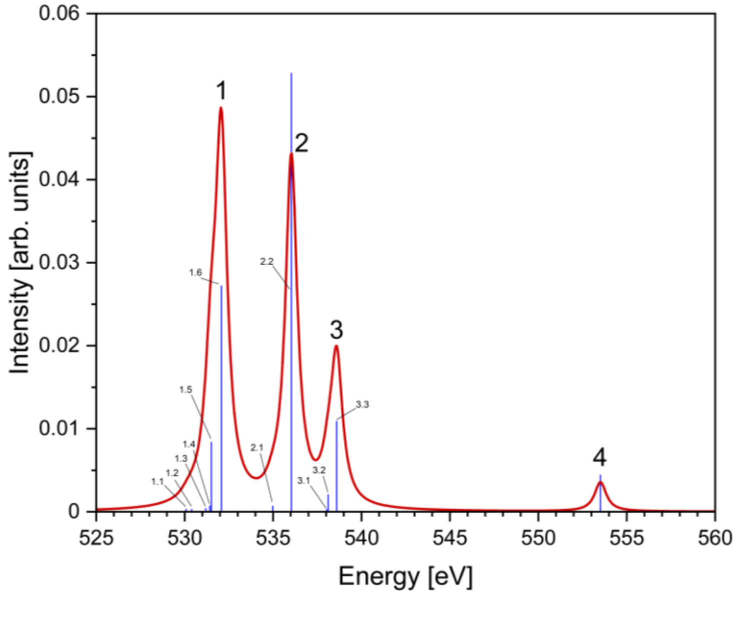

Figure S4: RAS(S) O K-edge XANES peak assignments for $[UO_2]^{2+}$. Assignments are reported in table S5. $[UO_2]^{2+}$ data taken from simulations reported in ref 1.

Table S5: RAS(S) $[UO_2]^{2+}$ peak assignments for figure S4. $[UO_2]^{2+}$ data taken from simulations reported in ref 1.

Transition	State	Energy	1s(g)	1s(u)	σg	πu	σu	πg	ϕu	δu	σu*	πu*	πg*	σg*
	GS	0	2.00	2.00	2.00	3.97	1.99	4.00	0.00	0.00	0.01	0.03	0.00	0.00
1.1	4/5	530.08	1.00	2.00	2.00	4.00	2.00	4.00	0.16	0.84	0.00	0.00	0.00	0.00
1.2	7/8	530.40	1.00	2.00	2.00	4.00	2.00	4.00	0.73	0.27	0.00	0.00	0.00	0.00
1.3	14/15	531.19	1.00	2.00	2.00	4.00	2.00	4.00	0.93	0.07	0.00	0.00	0.00	0.00
1.4	19	531.42	1.10	1.90	2.00	4.00	2.00	4.00	0.00	0.00	0.01	0.88	0.10	0.00
1.5	20/21	531.51	1.10	1.90	2.00	4.00	2.00	4.00	0.00	0.00	0.03	0.87	0.10	0.00
**1.6**	**24/25**	**532.08**	**1.09**	**1.91**	**2.00**	**4.00**	**2.00**	**4.00**	**0.00**	**0.05**	**0.00**	**0.86**	**0.09**	**0.00**
2.1	27/28	534.98	1.05	1.95	2.00	4.00	2.00	4.00	0.00	0.00	0.93	0.02	0.00	0.05
**2.2**	**29**	**536.03**	**0.05**	**1.95**	**2.00**	**4.00**	**2.00**	**4.00**	**0.00**	**0.00**	**0.95**	**0.00**	**0.00**	**0.04**
3.1	31	538.03	1.88	1.12	2.00	4.00	2.00	4.00	0.00	0.00	0.00	0.12	0.88	0.00
3.2	32/33	538.12	1.89	1.11	2.00	4.00	2.00	4.00	0.00	0.00	0.00	0.10	0.90	0.00
**3.3**	**36/37**	**538.60**	**1.91**	**1.09**	**2.00**	**4.00**	**2.00**	**4.00**	**0.00**	**0.00**	**0.00**	**0.10**	**0.90**	**0.00**
**4**	**41**	**553.51**	**1.96**	**1.04**	**2.00**	**4.00**	**2.00**	**4.00**	**0.00**	**0.00**	**0.04**	**0.00**	**0.00**	**0.96**

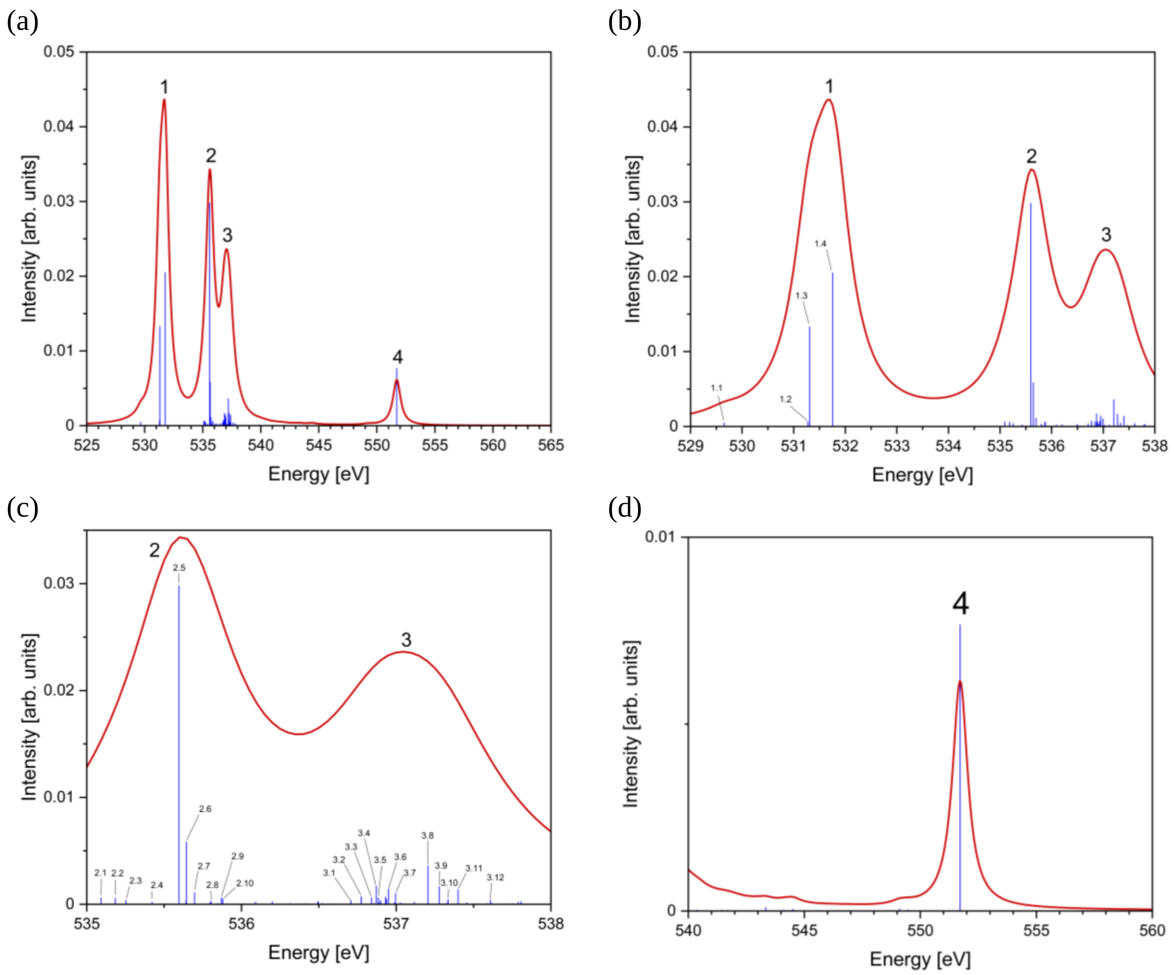

Figure S5: RAS(SD) O K-edge XANES peak assignments for $[UO_2]^{2+}$. Figures (a)-(d) present the spectrum and assignments at different energy scales. Assignments are contained in table S6. $[UO_2]^{2+}$ data taken from simulations reported in ref 1.

Table S6: RAS(SD) $[UO_2]^{2+}$ peak assignments for figure S5. Bolded values identify data chosen to be represented in the main text. $[UO_2]^{2+}$ data taken from simulations reported in ref 1.

Transition	State	Energy	1s(g)	1s(u)	σg	πu	σu	πg	ϕu	δu	σu*	πu*	πg*	σg*
	GS	0	2.00	2.00	1.97	3.92	1.97	3.95	0.00	0.00	0.05	0.08	0.05	0.01
**1.1**	**8/9**	**529.65**	**1.24**	**1.76**	**1.93**	**3.89**	**1.98**	**3.92**	**0.00**	**0.96**	**0.08**	**0.12**	**0.10**	**0.00**
1.2	19	531.28	1.27	1.73	1.93	3.89	1.98	3.92	0.00	0.02	0.09	1.00	0.18	0.00
1.3	20/21	531.31	1.27	1.73	1.93	3.89	1.98	3.92	0.00	0.02	0.09	1.00	0.16	0.00
**1.4**	**24/25**	**531.76**	**1.27**	**1.73**	**1.93**	**3.89**	**1.98**	**3.92**	**0.00**	**0.04**	**0.08**	**0.99**	**0.16**	**0.00**
2.1	60/61	535.09	1.36	1.64	1.92	3.75	1.88	3.77	0.19	0.71	0.50	0.19	0.08	0.01
2.2	67	535.18	1.46	1.54	1.93	3.58	1.89	3.62	0.46	1.26	0.03	0.20	0.02	0.00
2.3	71	535.25	1.42	1.58	1.76	3.80	1.62	3.82	0.60	1.26	0.01	0.12	0.00	0.00
2.4	80	535.42	1.43	1.57	1.76	3.79	1.64	3.81	0.54	1.23	0.01	0.22	0.00	0.00
**2.5**	**99**	**535.6**	**1.32**	**1.68**	**1.94**	**3.78**	**1.93**	**3.80**	**0.18**	**0.46**	**0.68**	**0.16**	**0.08**	**0.01**
2.6	109	535.64	1.44	1.56	1.92	3.62	1.88	3.66	0.45	1.12	0.13	0.18	0.04	0.00
2.7	118	535.7	1.44	1.56	1.80	3.75	1.71	3.77	0.58	1.10	0.04	0.25	0.02	0.00
2.8	129	535.8	1.44	1.56	1.78	3.76	1.67	3.78	0.67	1.04	0.01	0.28	0.02	0.00
2.9	145	535.87	1.46	1.54	1.95	3.55	1.92	3.60	0.76	1.06	0.02	0.12	0.02	0.00
2.10	148	535.88	1.47	1.53	1.96	3.54	1.94	3.58	0.78	1.04	0.02	0.12	0.02	0.00
3.1	296	536.71	1.45	1.55	1.82	3.73	1.72	3.75	0.60	0.54	0.03	0.77	0.04	0.00
3.2	309/310	536.77	1.47	1.53	1.87	3.65	1.81	3.68	0.52	0.53	0.03	1.14	0.07	0.00
3.3	317/318	536.84	1.47	1.53	1.85	3.69	1.78	3.72	0.61	0.45	0.04	0.75	0.11	0.00
3.4	324/325	536.87	1.49	1.51	1.89	3.66	1.83	3.69	0.63	0.47	0.04	0.64	0.16	0.00
3.5	327	536.88	1.46	1.54	1.91	3.60	1.86	3.64	0.52	0.58	0.03	0.83	0.04	0.00
3.6	342/343	536.95	1.49	1.51	1.93	3.59	1.90	3.63	0.43	0.57	0.04	0.80	0.12	0.00
3.7	352/353	537	1.48	1.52	1.88	3.67	1.82	3.70	0.41	0.60	0.04	0.74	0.15	0.00
**3.8**	**395/396**	**537.21**	**1.52**	**1.48**	**1.91**	**3.65**	**1.89**	**3.68**	**0.65**	**0.37**	**0.05**	**0.54**	**0.26**	**0.00**
3.9	406/407	537.28	1.49	1.51	1.94	3.58	1.92	3.62	0.86	0.33	0.03	0.62	0.11	0.00
3.10	419/420	537.33	1.48	1.52	1.91	3.66	1.88	3.69	0.61	0.47	0.05	0.48	0.26	0.00
3.11	433/434	537.4	1.49	1.51	1.95	3.56	1.93	3.60	0.56	0.49	0.02	0.75	0.12	0.00
3.12	480/481	537.61	1.46	1.54	1.85	3.69	1.78	3.72	0.48	0.61	0.05	0.73	0.09	0.00
**4**	**1377**	**551.71**	**1.74**	**1.26**	**1.92**	**3.88**	**1.97**	**3.84**	**0.00**	**0.00**	**0.13**	**0.22**	**0.08**	**0.97**

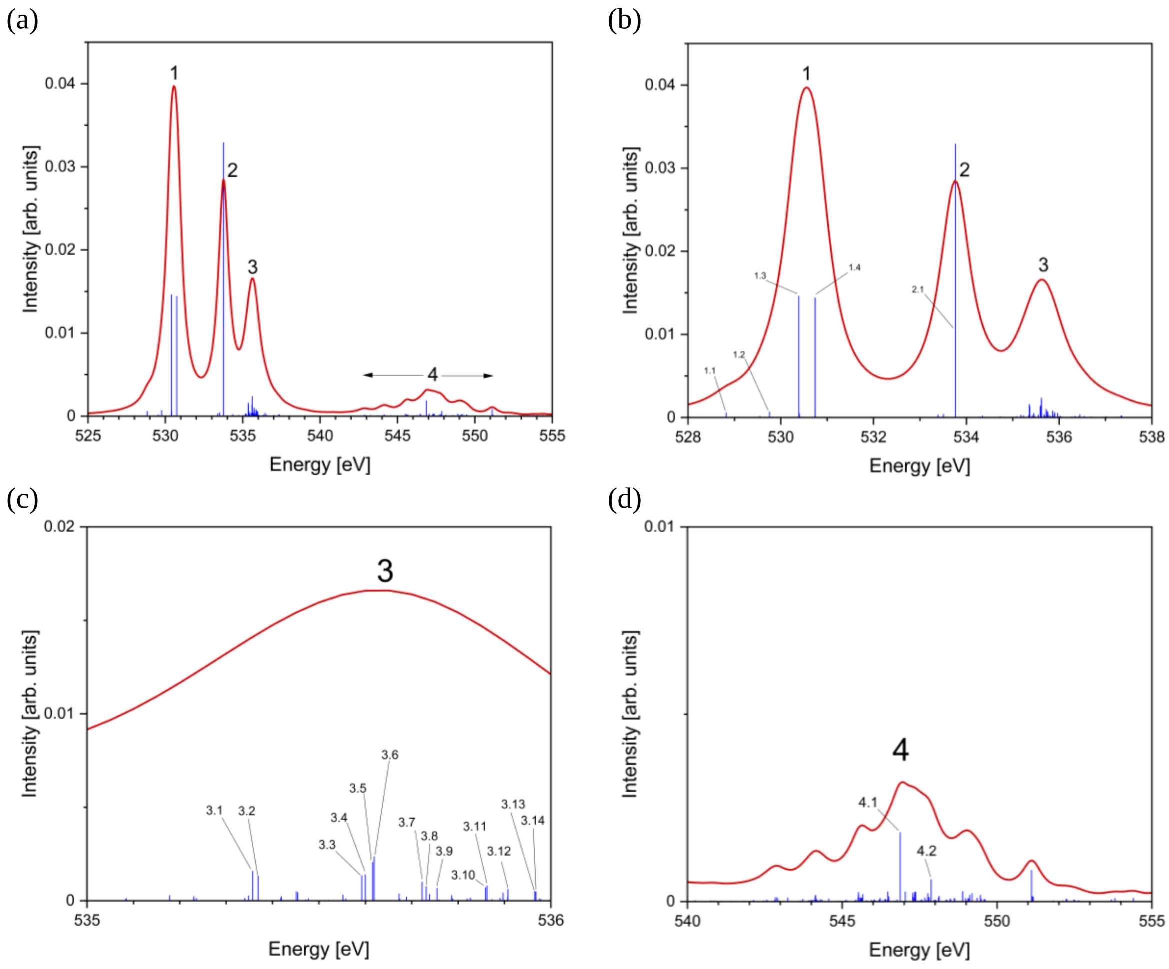

Figure S6: RAS(SD) O K-edge XANES peak assignments for $[UO_2Cl_4]^{2-}$. Figures (a)-(d) present the spectrum and assignments at different energy scales. Assignments are contained in table S7.

Table S7: RAS(SD) $[UO_2Cl_4]^{2-}$ peak assignments for figure S6. Bolded values identify data chosen to be represented in the main text.

Transition	State	Energy	1s(g)	1s(u)	σg	πu	σu	πg	ϕu	δu	σu*	πu*	πg*	σg*
	GS	0	2.00	2.00	1.97	3.90	1.96	3.94	0.00	0.01	0.06	0.10	0.05	0.00
1.1	4/5	528.82	1.24	1.76	1.93	3.89	1.98	3.92	0.24	0.77	0.08	0.07	0.12	0.00
1.2	14/15	529.76	1.24	1.76	1.94	3.89	1.98	3.91	0.98	0.02	0.08	0.09	0.11	0.00
**1.3**	**18/19**	**530.39**	**1.26**	**1.74**	**1.94**	**3.88**	**1.98**	**3.92**	**0.01**	**0.01**	**0.09**	**1.00**	**0.17**	**0.00**
1.4	22/23	530.74	1.27	1.73	1.94	3.88	1.98	3.92	0.01	0.05	0.07	0.98	0.18	0.00
**2.1**	**36**	**533.76**	**1.27**	**1.73**	**1.96**	**3.85**	**1.97**	**3.86**	**0.02**	**0.08**	**0.95**	**0.17**	**0.13**	**0.00**
3.1	93	535.36	1.45	1.55	1.78	3.82	1.64	3.84	0.45	0.60	0.04	0.64	0.18	0.00
3.2	94	535.37	1.45	1.55	1.75	3.88	1.58	3.89	0.52	0.47	0.05	0.67	0.20	0.00
3.3	115	535.59	1.45	1.55	1.82	3.76	1.69	3.79	0.49	0.75	0.02	0.55	0.13	0.00
3.4	116	535.6	1.45	1.55	1.82	3.76	1.69	3.79	0.49	0.75	0.03	0.54	0.13	0.00
3.5	119	535.62	1.46	1.54	1.77	3.84	1.63	3.85	0.37	0.61	0.04	0.70	0.19	0.00
**3.6**	**120**	**535.62**	**1.47**	**1.53**	**1.79**	**3.82**	**1.65**	**3.83**	**0.40**	**0.57**	**0.04**	**0.69**	**0.20**	**0.00**
3.7	132	535.72	1.49	1.51	1.80	3.82	1.68	3.83	0.30	0.36	0.05	0.86	0.29	0.00
3.8	133	535.73	1.46	1.54	1.78	3.84	1.63	3.85	0.38	0.36	0.05	0.90	0.22	0.00

3.9	138	535.75	1.42	1.58	1.73	3.87	1.55	3.88	0.55	0.49	0.04	0.78	0.10	0.00
3.10	151	535.86	1.45	1.55	1.83	3.74	1.71	3.77	0.58	0.80	0.02	0.46	0.09	0.00
3.11	152	535.86	1.45	1.55	1.83	3.75	1.70	3.77	0.66	0.70	0.03	0.45	0.12	0.00
3.12	162	535.91	1.42	1.58	1.74	3.86	1.56	3.88	0.76	0.66	0.03	0.41	0.11	0.00
3.13	167	535.97	1.46	1.54	1.87	3.68	1.78	3.70	0.58	0.98	0.02	0.35	0.06	0.00
3.14	168	535.97	1.46	1.54	1.88	3.66	1.80	3.68	0.58	0.97	0.02	0.36	0.05	0.00
4.1	2318	546.87	1.54	1.46	1.80	3.70	1.85	3.66	0.13	0.10	1.05	0.50	0.19	0.02
**4.2**	**2435**	**547.87**	**1.72**	**1.29**	**1.94**	**3.86**	**1.97**	**3.81**	**0.01**	**0.01**	**0.10**	**0.27**	**0.06**	**0.98**

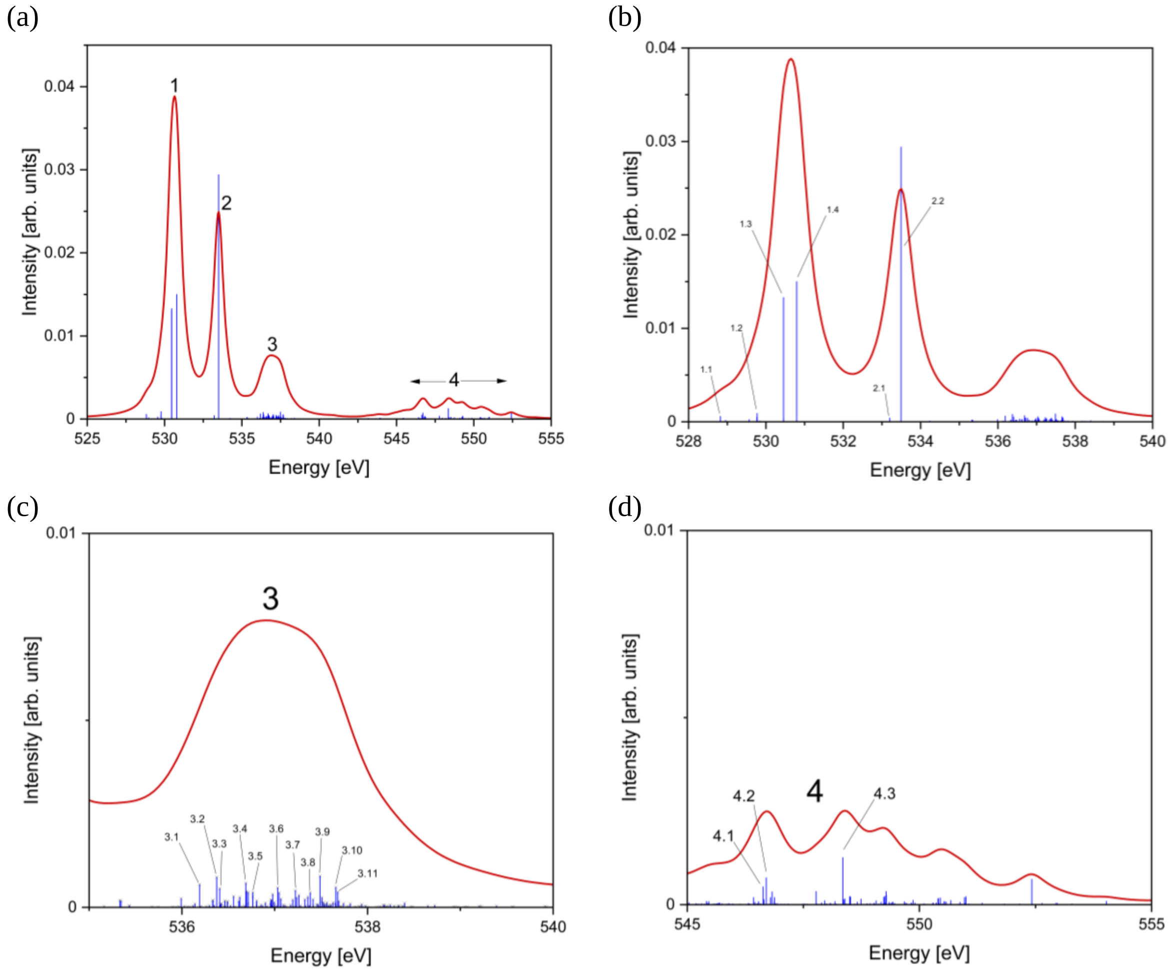

Figure S7: RAS(SD) O K-edge XANES peak assignments for $Cs_2UO_2Cl_4$. Figures (a)-(d) present the spectrum and assignments at different energy scales. Assignments are contained in table S8.

Table S8: RAS(SD) $Cs_2UO_2Cl_4$ peak assignments for figure S7. Bolded values identify data chosen to be represented in the main text.

Transition	State	Energy	1s(g)	1s(u)	σg	πu	σu	πg	σu*	πu*	πg*	σg*	ϕu	δu
	GS	0	2.00	2.00	1.97	3.92	1.96	3.95	0.06	0.09	0.04	0.01	0.00	0.01
1.1	4/5	528.82	1.23	1.77	1.94	3.88	1.99	3.93	0.07	0.06	0.12	0.00	0.24	0.77
1.2	14/15	529.77	1.23	1.77	1.94	3.88	1.99	3.92	0.07	0.08	0.12	0.00	0.98	0.02
1.3	19/20	530.45	1.26	1.74	1.95	3.88	1.98	3.93	0.08	0.98	0.18	0.00	0.01	0.01
**1.4**	**24/25**	**530.8**	**1.26**	**1.74**	**1.95**	**3.88**	**1.98**	**3.93**	**0.06**	**0.96**	**0.18**	**0.00**	**0.01**	**0.05**
2.1	27/28	533.21	1.24	1.76	1.97	3.86	1.98	3.89	0.98	0.14	0.14	0.00	0.00	0.05
**2.2**	**29**	**533.49**	**1.25**	**1.75**	**1.96**	**3.85**	**1.98**	**3.89**	**0.97**	**0.14**	**0.14**	**0.00**	**0.01**	**0.06**
**3.1**	**92**	**536.19**	**1.47**	**1.54**	**1.83**	**3.78**	**1.70**	**3.80**	**0.04**	**0.42**	**0.22**	**0.00**	**0.48**	**0.73**
3.2	111	536.37	1.43	1.57	1.79	3.81	1.62	3.83	0.03	0.48	0.11	0.00	0.62	0.71
3.3	112	536.41	1.44	1.56	1.85	3.72	1.72	3.75	0.02	0.34	0.10	0.00	0.61	0.90
3.4	147	536.69	1.45	1.55	1.84	3.74	1.72	3.77	0.02	0.46	0.14	0.00	0.62	0.70
3.5	158	536.77	1.44	1.56	1.89	3.64	1.80	3.69	0.01	0.29	0.05	0.00	0.71	0.91
3.6	190	537.03	1.43	1.57	1.80	3.78	1.64	3.81	0.04	0.61	0.10	0.00	0.54	0.68
3.7	213	537.22	1.44	1.56	1.88	3.67	1.77	3.71	0.05	0.54	0.07	0.00	0.59	0.73
3.8	236	537.38	1.45	1.55	1.90	3.63	1.82	3.68	0.08	0.53	0.07	0.00	0.48	0.82
3.9	257	537.49	1.47	1.53	1.92	3.61	1.87	3.65	0.04	0.58	0.13	0.00	0.68	0.53
3.10	293	537.66	1.47	1.53	1.93	3.59	1.88	3.63	0.03	0.42	0.08	0.00	0.53	0.91
3.11	297	537.68	1.45	1.55	1.90	3.63	1.82	3.68	0.07	0.66	0.08	0.00	0.49	0.67
4.1	2092	546.63	1.48	1.52	1.73	3.75	1.82	3.71	0.20	0.40	0.75	0.01	0.33	0.30
4.2	2097	546.7	1.51	1.49	1.70	3.82	1.78	3.73	0.29	0.59	0.61	0.02	0.22	0.23
4.3	2333	548.35	1.52	1.48	1.87	3.63	1.92	3.58	0.69	0.48	0.38	0.02	0.13	0.28

4. Spin-Free uranyl O K-edge XANES spectra

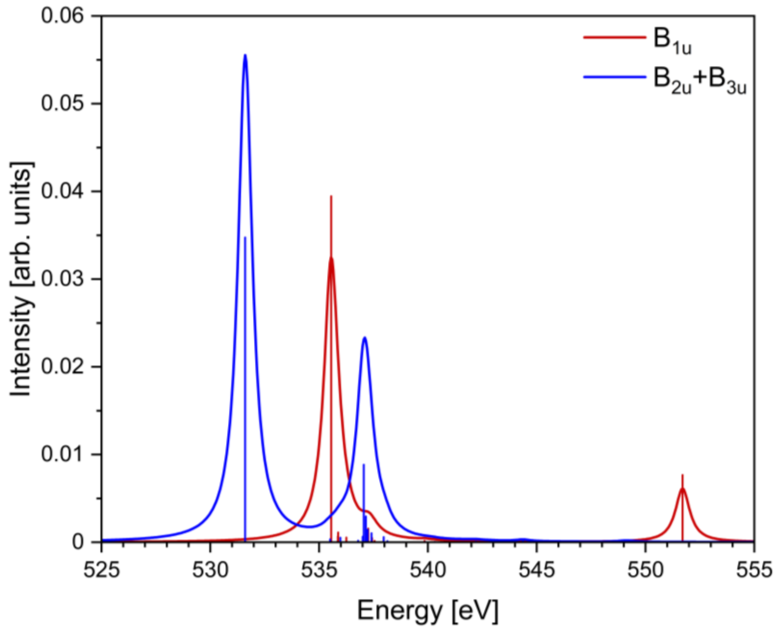

Figure S8: Spin-Free RAS(SD) O K-edge XANES for $[UO_2]^{2+}$ showing peaks are generated by transitions belonging to B1u or B2u/B3u transition symmetries. Peaks align with X-ray photons which are incident parallel (B1u) or perpendicular (B2u/B3u) to the O-U-O axis. $[UO_2]^{2+}$ data taken from simulations reported in ref 1.

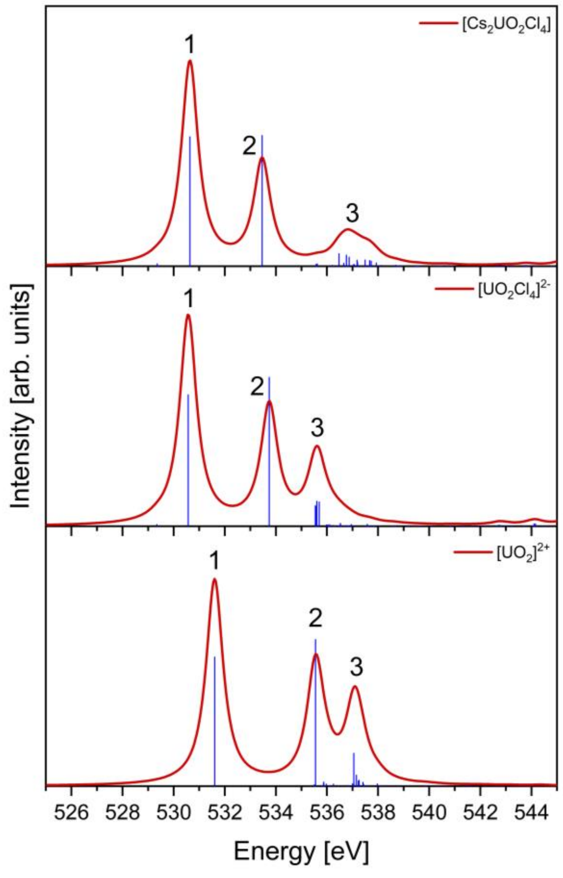

Figure S9: Spin-Free RAS(SD) O K-edge XANES for $[UO_2]^{2+}$, $[UO_2Cl_4]^{2-}$ and $Cs_2UO_2Cl_4$. $[UO_2]^{2+}$ data taken from simulations reported in ref 1.

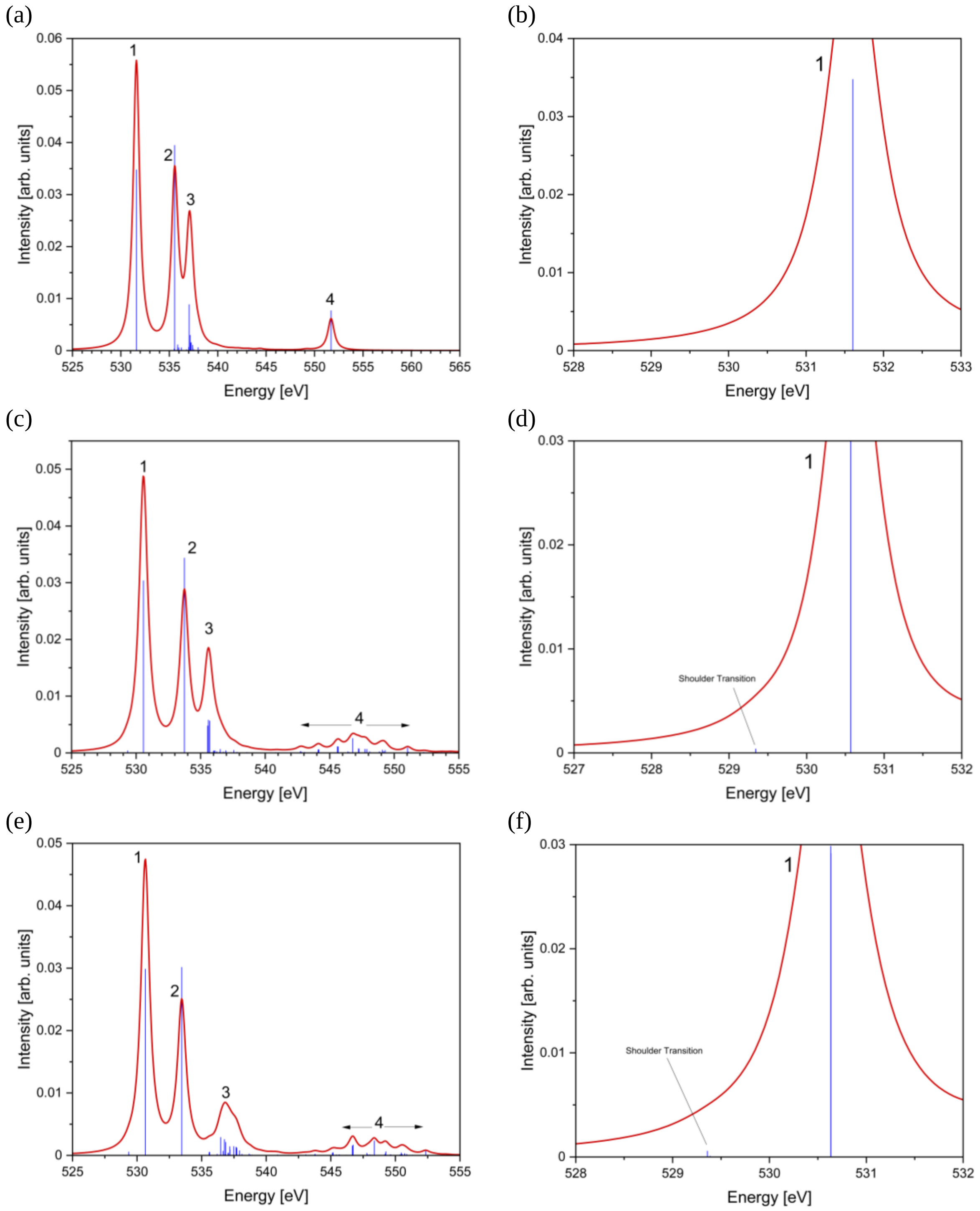

Figure S10: Spin-Free RAS(SD) O K-edge XANES spectra for (a-b) $[UO_2]^{2+}$, (c-d) $[UO_2Cl_4]^{2-}$ and (e-f) $Cs_2UO_2Cl_4$. Figures b,d, and f, show the absence or presence of a shoulder transition. $[UO_2]^{2+}$ data taken from simulations reported in ref 1.

5. QTAIM Metrics:

Table S9: GS and CES QTAIM metrics for the three different uranyl models. Table reports the U-O delocalisation indexes $\delta(\mathrm{U,O})$ as well as the uranium and oxygen localisation indexes $\lambda(\mathrm{U})$ and $\lambda(\mathrm{O})$. Analysis is from RAS(SD) electron densities. CESs chosen for analysis correspond to those assigned in Table 3 of the main manuscript. Additional data is reported in Tables S10-12. $[UO_2]^{2+}$ data taken from simulations reported in ref 1.

Model	Property	GS	$1s \longrightarrow \pi_u^*$	$1s \longrightarrow \sigma_u^*$	$1s \longrightarrow \pi_g^*$
$[UO_2]^{2+}$ [1]	δ(U,O)	1.85	1.30	1.23	1.17
	λ(U)	86.85	87.56	87.68	87.83
	λ(O)	7.70	7.90	7.91	7.91
$[UO_2Cl_4]^{2-}$	δ(U,O)	1.53	1.10	1.10	0.96
	λ(U)	86.37	87.06	87.06	87.36
	λ(O)	8.03	8.12	8.13	8.16
$Cs_2UO_2Cl_4$	δ(U,O)	1.52	1.11	1.10	0.97
	λ(U)	86.35	87.04	87.01	87.36
	λ(O)	8.06	8.14	8.15	8.17

Table S10: QTAIM analysis data for $[UO_2]^{2+}$ RAS(SD) states. Bolded values identify data chosen to be represented in the main text. $[UO_2]^{2+}$ data taken from simulations reported in ref 1.

	GS	**1s → πu*<br>Transition 1.4<br>State 24**	**Δ**	**1s → σu*<br>Transition 2.5<br>State 99**	**Δ**	**1s → πg*<br>Transition 3.8<br>State 395**	**Δ**	1s → σg*<br>Transition 4<br>State 1377	Δ
ρ_{BCP}(U-O)	0.33	**0.31**	**-0.03**	**0.32**	**-0.02**	**0.29**	**-0.04**	0.32	-0.01
δ(U,O)	1.85	**1.30**	**-0.55**	**1.23**	**-0.62**	**1.17**	**-0.68**	1.46	-0.39
δ(O,O)	0.06	**0.04**	**-0.01**	**0.03**	**-0.02**	**0.02**	**-0.04**	0.09	0.03
λ(U)	86.85	**87.56**	**0.71**	**87.68**	**0.83**	**87.83**	**0.99**	87.38	0.53
λ(O1)	7.70	**7.90**	**0.20**	**7.91**	**0.22**	**7.91**	**0.21**	7.79	0.09
λ(O2)	7.70	**7.90**	**0.20**	**7.91**	**0.22**	**7.91**	**0.21**	7.79	0.09
BCP (X,Y,Z)	1.85	**1.81**	**-0.03**	**1.88**	**0.03**	**1.79**	**-0.05**	1.88	0.03
q(U)	3.30	**3.14**	**-0.16**	**3.09**	**-0.21**	**3.00**	**-0.30**	3.15	-0.15
q(O1)	-0.65	**-0.57**	**0.08**	**-0.55**	**0.10**	**-0.50**	**0.15**	-0.57	0.08
q(O2)	-0.65	**-0.57**	**0.08**	**-0.55**	**0.10**	**-0.50**	**0.15**	-0.57	0.08

Table S11: QTAIM analysis data for $[UO_2Cl_4]^{2-}$ RAS(SD) states. Bolded values identify data chosen to be represented in the main text.

		**Peak 1**		Peak 1		**Peak 2**		Peak 3		**Peak 3**		Peak 3		Peak 3		Peak 4	
	GS	**Transition 1.3**	**Δ**	Transition 1.4	Δ	**Transition 2.1**	**Δ**	Transition 3.2	Δ	**Transition 3.6**	**Δ**	Transition 3.7	Δ	Transition 3.8	Δ	Transition 4.2	Δ
		**State 18**		State 22		**State 36**		State 94		**State 120**		State 132		State 133		State 2435	
ρ_{BCP}(U-O)	0.31	**0.28**	**-0.03**	0.28	-0.03	**0.31**	**0.00**	0.26	-0.05	**0.26**	**-0.05**	0.27	-0.05	0.26	-0.05	0.29	-0.03
ρ_{BCP}(U-Cl)	0.06	**0.06**	**0.00**	0.06	0.00	**0.06**	**0.00**	0.06	0.00	**0.06**	**0.00**	0.06	0.00	0.06	0.00	0.06	0.00
δ(U,O1)	1.53	**1.10**	**-0.42**	1.11	-0.42	**1.10**	**-0.43**	0.94	-0.59	**0.96**	**-0.57**	0.95	-0.58	0.94	-0.59	1.15	-0.38
δ(U,O2)	1.53	**1.10**	**-0.42**	1.11	-0.42	**1.10**	**-0.43**	0.94	-0.59	**0.96**	**-0.57**	0.95	-0.58	0.94	-0.59	1.15	-0.38
δ(U,Cl1)	0.40	**0.40**	**0.01**	0.41	0.01	**0.41**	**0.01**	0.40	0.01	**0.41**	**0.01**	0.40	0.01	0.40	0.01	0.44	0.04
δ(U,Cl2)	0.40	**0.41**	**0.01**	0.41	0.01	**0.41**	**0.01**	0.40	0.01	**0.41**	**0.01**	0.40	0.01	0.40	0.01	0.44	0.04
δ(U,Cl3)	0.39	**0.40**	**0.01**	0.40	0.01	**0.41**	**0.01**	0.40	0.01	**0.40**	**0.00**	0.41	0.02	0.40	0.00	0.44	0.05
δ(U,Cl4)	0.39	**0.40**	**0.01**	0.40	0.01	**0.41**	**0.01**	0.40	0.01	**0.40**	**0.00**	0.41	0.02	0.40	0.00	0.44	0.04
λ(U)	86.37	**87.06**	**0.69**	87.06	0.69	**87.06**	**0.68**	87.35	0.98	**87.36**	**0.98**	87.33	0.96	87.36	0.98	86.38	0.00
λ(O1)	8.03	**8.12**	**0.09**	8.12	0.09	**8.13**	**0.10**	8.19	0.15	**8.16**	**0.13**	8.17	0.14	8.18	0.15	7.98	-0.05
λ(O2)	8.03	**8.12**	**0.09**	8.12	0.09	**8.13**	**0.10**	8.19	0.15	**8.16**	**0.13**	8.17	0.14	8.18	0.15	7.98	-0.05
λ(Cl1)	17.54	**17.55**	**0.01**	17.55	0.01	**17.55**	**0.00**	17.54	0.00	**17.54**	**0.00**	17.55	0.00	17.54	0.00	17.59	0.04
λ(Cl2)	17.54	**17.55**	**0.01**	17.55	0.01	**17.55**	**0.00**	17.54	0.00	**17.54**	**0.00**	17.55	0.00	17.54	0.00	17.59	0.04
λ(Cl3)	17.55	**17.55**	**0.00**	17.55	0.00	**17.55**	**0.00**	17.54	0.00	**17.54**	**0.00**	17.55	0.00	17.55	0.00	17.59	0.04
λ(Cl4)	17.55	**17.55**	**0.00**	17.55	0.00	**17.55**	**0.00**	17.54	0.00	**17.54**	**0.00**	17.55	0.00	17.55	0.00	17.59	0.04
q(U)	3.31	**3.02**	**-0.29**	3.02	-0.29	**3.03**	**-0.28**	2.90	-0.41	**2.88**	**-0.43**	2.90	-0.41	2.90	-0.41	3.59	0.28
q(O1)	-0.93	**-0.78**	**0.15**	-0.78	0.15	**-0.79**	**0.14**	-0.75	0.18	**-0.73**	**0.20**	-0.74	0.19	-0.74	0.19	-0.69	0.24
q(O2)	-0.93	**-0.78**	**0.15**	-0.78	0.15	**-0.79**	**0.14**	-0.75	0.18	**-0.73**	**0.20**	-0.74	0.19	-0.74	0.19	-0.69	0.24
q(Cl1)	-0.86	**-0.86**	**0.00**	-0.86	0.00	**-0.86**	**0.00**	-0.85	0.01	**-0.85**	**0.01**	-0.85	0.01	-0.85	0.01	-1.05	-0.19
q(Cl2)	-0.86	**-0.86**	**0.00**	-0.86	0.00	**-0.86**	**0.00**	-0.85	0.01	**-0.85**	**0.01**	-0.85	0.01	-0.85	0.01	-1.05	-0.19
q(Cl3)	-0.86	**-0.86**	**0.00**	-0.86	0.00	**-0.86**	**0.00**	-0.85	0.01	**-0.85**	**0.01**	-0.86	0.00	-0.85	0.01	-1.05	-0.19
q(Cl4)	-0.86	**-0.86**	**0.00**	-0.86	0.00	**-0.86**	**0.00**	-0.85	0.01	**-0.85**	**0.01**	-0.86	0.00	-0.85	0.01	-1.05	-0.19

Table S12: QTAIM analysis data for $Cs_2UO_2Cl_4$ RAS(SD) states. Bolded values identify data chosen to be represented in the main text.

	GS	Peak 1<br>Transition 1.3<br>State 19	Δ	**Peak 1<br>Transition 1.4<br>State 24**	**Δ**	**Peak 2<br>Transition 2.2<br>State 29**	**Δ**	**Peak 3<br>Transition 3.1<br>State 92**	**Δ**	Peak 3<br>Transition 3.2<br>State 111	Δ	Peak 3<br>Transition 3.4<br>State 147	Δ	Peak 3<br>Transition 3.6<br>State 190	Δ	Peak 3<br>Transition 3.9<br>State 257	Δ
ρ_{BCP}(U-O)	0.32	0.28	-0.04	**0.28**	**-0.04**	**0.31**	**-0.01**	**0.26**	**-0.05**	0.26	-0.06	0.26	-0.05	0.26	-0.05	0.27	-0.05
ρ_{BCP}(U-Cl)	0.06	0.06	0.00	**0.06**	**0.00**	**0.06**	**0.00**	**0.06**	**0.00**	0.06	0.00	0.06	0.00	0.06	0.00	0.06	0.00
δ(U,O1)	1.52	1.10	-0.42	**1.11**	**-0.42**	**1.10**	**-0.42**	**0.97**	**-0.55**	0.96	-0.57	0.96	-0.56	0.94	-0.58	0.95	-0.57
δ(U,O2)	1.52	1.10	-0.42	**1.11**	**-0.42**	**1.10**	**-0.42**	**0.97**	**-0.55**	0.96	-0.57	0.96	-0.56	0.94	-0.58	0.95	-0.57
δ(U,Cl1)	0.41	0.42	0.01	**0.42**	**0.01**	**0.43**	**0.02**	**0.41**	**0.00**	0.41	-0.01	0.41	0.00	0.40	-0.01	0.41	0.00
δ(U,Cl2)	0.41	0.42	0.01	**0.42**	**0.01**	**0.43**	**0.02**	**0.41**	**0.00**	0.41	-0.01	0.41	0.00	0.40	-0.01	0.41	0.00
δ(U,Cl3)	0.41	0.42	0.01	**0.42**	**0.01**	**0.43**	**0.02**	**0.41**	**0.00**	0.41	-0.01	0.41	0.00	0.40	-0.01	0.41	0.00
δ(U,Cl4)	0.41	0.42	0.01	**0.42**	**0.01**	**0.43**	**0.02**	**0.41**	**0.00**	0.41	-0.01	0.41	0.00	0.40	-0.01	0.41	0.00
λ(U)	86.35	87.04	0.69	**87.04**	**0.69**	**87.01**	**0.66**	**87.36**	**1.00**	87.39	1.03	87.40	1.04	87.41	1.06	87.44	1.09
λ(O1)	8.06	8.14	0.09	**8.14**	**0.09**	**8.15**	**0.10**	**8.17**	**0.11**	8.18	0.12	8.16	0.10	8.18	0.12	8.14	0.09
λ(O2)	8.06	8.14	0.09	**8.14**	**0.09**	**8.15**	**0.10**	**8.17**	**0.11**	8.18	0.12	8.16	0.10	8.18	0.12	8.14	0.09
λ(Cl1)	17.52	17.52	0.00	**17.52**	**0.00**	**17.51**	**-0.01**	**17.52**	**0.00**	17.52	0.00	17.52	0.00	17.52	0.00	17.52	0.00
λ(Cl2)	17.52	17.52	0.00	**17.52**	**0.00**	**17.51**	**-0.01**	**17.52**	**0.00**	17.52	0.00	17.52	0.00	17.52	0.00	17.52	0.00
λ(Cl3)	17.52	17.52	0.00	**17.52**	**0.00**	**17.51**	**-0.01**	**17.52**	**0.00**	17.52	0.00	17.52	0.00	17.52	0.00	17.52	0.00
λ(Cl4)	17.52	17.52	0.00	**17.52**	**0.00**	**17.51**	**-0.01**	**17.52**	**0.00**	17.52	0.00	17.52	0.00	17.52	0.00	17.52	0.00
q(U)	3.30	3.01	-0.29	**3.01**	**-0.29**	**3.02**	**-0.28**	**2.85**	**-0.45**	2.84	-0.45	2.82	-0.47	2.84	-0.46	2.78	-0.51
q(O1)	-0.95	-0.81	0.14	**-0.81**	**0.14**	**-0.83**	**0.13**	**-0.75**	**0.20**	-0.75	0.20	-0.74	0.22	-0.75	0.20	-0.71	0.24
q(O2)	-0.95	-0.81	0.14	**-0.81**	**0.14**	**-0.83**	**0.13**	**-0.75**	**0.20**	-0.75	0.20	-0.74	0.22	-0.75	0.20	-0.71	0.24
q(Cl1)	-0.85	-0.85	0.00	**-0.85**	**0.00**	**-0.84**	**0.01**	**-0.84**	**0.01**	-0.83	0.01	-0.84	0.01	-0.83	0.01	-0.84	0.01
q(Cl2)	-0.85	-0.85	0.00	**-0.85**	**0.00**	**-0.84**	**0.01**	**-0.84**	**0.01**	-0.83	0.01	-0.84	0.01	-0.83	0.01	-0.84	0.01
q(Cl3)	-0.85	-0.85	0.00	**-0.85**	**0.00**	**-0.84**	**0.01**	**-0.84**	**0.01**	-0.83	0.01	-0.84	0.01	-0.83	0.01	-0.84	0.01
q(Cl4)	-0.85	-0.85	0.00	**-0.85**	**0.00**	**-0.84**	**0.01**	**-0.84**	**0.01**	-0.83	0.01	-0.84	0.01	-0.83	0.01	-0.84	0.01

6. Orbital Composition Analysis:

Table S13: Atoms in molecules (AIM) percentage composition analysis for $[UO_2]^{2+}$ SONOs in RAS(SD) states. Bolded values identify data chosen to be represented in the main text. $[UO_2]^{2+}$ data taken from simulations reported in ref 1.

	Atom	σg	πu		σu	πg		σu*	πu*		πg*		σg*
Peak0: Transition 0: Ground-State	U	18.6	25.1	25.1	53.4	17.2	17.2	50.2	73.5	73.5	45.1	45.1	48.5
	O	40.7	37.4	37.4	23.3	41.4	41.4	24.9	13.2	13.2	27.5	27.5	25.8
	O	40.7	37.4	37.4	23.3	41.4	41.4	24.9	13.2	13.2	27.5	27.5	25.8
Peak 1 Transition 1.3: State 20	U	13.4	15.4	15.3	49.3	13.5	13.7	64.6	84.9	85.1	70.2	70.3	64.7
	O	43.3	42.3	42.4	25.4	43.3	43.2	17.7	7.6	7.4	14.9	14.9	17.7
	O	43.3	42.3	42.4	25.4	43.3	43.2	17.7	7.6	7.4	14.9	14.9	17.7
**Peak 1 Transition 1.4: State 24**	**U**	**13.3**	**15.0**	**15.2**	**49.3**	**13.4**	**13.6**	**64.7**	**85.4**	**85.0**	**70.2**	**70.4**	**64.6**
	**O**	**43.3**	**42.5**	**42.4**	**25.4**	**43.3**	**43.2**	**17.7**	**7.3**	**7.5**	**14.9**	**14.8**	**17.7**
	**O**	**43.3**	**42.5**	**42.4**	**25.4**	**43.3**	**43.2**	**17.7**	**7.3**	**7.5**	**14.9**	**14.8**	**17.7**
**Peak 2 Transition 2.5: State 99**	**U**	**14.2**	**13.9**	**13.9**	**46.3**	**11.9**	**11.9**	**68.2**	**86.3**	**86.4**	**71.5**	**71.5**	**63.5**
	**O**	**42.9**	**43.1**	**43.1**	**26.9**	**44.1**	**44.1**	**15.9**	**6.8**	**6.8**	**14.3**	**14.3**	**18.2**
	**O**	**42.9**	**43.1**	**43.1**	**26.9**	**44.1**	**44.1**	**15.9**	**6.8**	**6.8**	**14.3**	**14.3**	**18.2**
Peak 3 Transition 3.4: State 324	U	13.9	10.8	10.6	43.3	9.0	9.1	70.7	92.6	91.8	74.7	74.5	63.8
	O	43.1	44.6	44.7	28.3	45.5	45.5	14.6	3.7	4.1	12.7	12.8	18.1
	O	43.1	44.6	44.7	28.3	45.5	45.5	14.6	3.7	4.1	12.7	12.8	18.1
Peak 3 Transition 3.6: State 342	U	13.9	10.3	10.5	42.9	9.1	9.0	71.7	91.8	90.0	74.4	75.0	63.8
	O	43.0	44.8	44.8	28.6	45.5	45.5	14.2	4.1	5.0	12.8	12.5	18.1
	O	43.0	44.8	44.8	28.6	45.5	45.5	14.2	4.1	5.0	12.8	12.5	18.1
**Peak 3 Transition 3.8: State 395**	**U**	**13.8**	**11.7**	**11.4**	**44.1**	**9.4**	**9.2**	**69.8**	**91.5**	**89.6**	**74.1**	**75.2**	**63.9**
	**O**	**43.1**	**44.2**	**44.3**	**28.0**	**45.3**	**45.4**	**15.1**	**4.3**	**5.2**	**13.0**	**12.4**	**18.1**
	**O**	**43.1**	**44.2**	**44.3**	**28.0**	**45.3**	**45.4**	**15.1**	**4.3**	**5.2**	**13.0**	**12.4**	**18.0**
Peak 3 Transition 3.9: State 406	U	13.8	10.4	10.4	42.7	8.9	8.9	71.1	91.4	90.2	74.3	74.8	63.6
	O	43.1	44.8	44.8	28.7	45.6	45.6	14.4	4.3	4.9	12.9	12.6	18.2
	O	43.1	44.8	44.8	28.7	45.6	45.6	14.4	4.3	4.9	12.9	12.6	18.2
Peak 3 Transition 3.10: State 419	U	13.8	11.2	11.7	44.0	9.6	9.2	70.5	94.3	91.3	74.1	75.0	63.9
	O	43.1	44.4	44.2	28.0	45.2	45.4	14.7	2.9	4.3	13.0	12.5	18.1
	O	43.1	44.4	44.2	28.0	45.2	45.4	14.7	2.9	4.3	13.0	12.5	18.1
Peak 4 Transition 4: State 1377	U	14.4	17.8	17.8	51.6	11.9	11.9	63.2	82.9	82.9	73.2	73.2	61.0
	O	42.8	41.1	41.1	24.2	44.0	44.0	18.4	8.5	8.5	13.4	13.4	19.5
	O	42.8	41.1	41.1	24.2	44.0	44.0	18.4	8.5	8.5	13.4	13.4	19.5

Table S14: Atoms in molecules (AIM) percentage composition analysis for $[UO_2Cl_4]^{2-}$ SONOs in RAS(SD) states. Bolded values identify data chosen to be represented in the main text.

	Atom	σg	πu		σu	πg		σu*	πu*		πg*		σg*
	U	12.4	19.9	19.9	48.3	12.1	12.1	58.3	77.0	77.0	52.8	52.8	11.0
	O	43.3	39.5	39.5	24.9	42.2	42.2	20.8	11.1	11.1	19.6	19.6	5.9
	O	43.3	39.5	39.5	24.9	42.2	42.2	20.8	11.1	11.1	19.6	19.6	5.9
Peak0: Transition 0: Ground-State	Cl	0.1	0.1	0.5	0.4	1.6	0.1	0.0	0.0	0.3	3.5	0.5	19.3
	Cl	0.1	0.1	0.5	0.4	1.6	0.1	0.0	0.0	0.3	3.5	0.5	19.3
	Cl	0.3	0.5	0.1	0.4	0.1	1.6	0.0	0.3	0.0	0.5	3.5	19.3
	Cl	0.3	0.5	0.1	0.4	0.1	1.6	0.0	0.3	0.0	0.5	3.5	19.3
	**U**	**8.6**	**13.1**	**13.2**	**40.8**	**10.5**	**10.5**	**68.1**	**85.8**	**85.8**	**60.5**	**60.5**	**11.4**
	**O**	**45.3**	**43.0**	**43.0**	**23.1**	**43.3**	**43.3**	**15.3**	**6.2**	**6.3**	**12.9**	**12.8**	**5.4**
	**O**	**45.3**	**43.0**	**43.0**	**23.1**	**43.3**	**43.3**	**15.3**	**6.2**	**6.3**	**12.9**	**12.8**	**5.4**
**Peak 1 Transition 1.3: State 18**	**Cl**	**0.1**	**0.0**	**0.4**	**3.2**	**1.4**	**0.1**	**0.3**	**0.0**	**0.8**	**6.1**	**0.8**	**19.5**
	**Cl**	**0.1**	**0.0**	**0.4**	**3.2**	**1.4**	**0.1**	**0.3**	**0.0**	**0.8**	**6.1**	**0.8**	**19.5**
	**Cl**	**0.3**	**0.4**	**0.0**	**3.3**	**0.1**	**1.4**	**0.3**	**0.8**	**0.0**	**0.8**	**6.1**	**19.4**
	**Cl**	**0.3**	**0.4**	**0.0**	**3.3**	**0.1**	**1.4**	**0.3**	**0.8**	**0.0**	**0.8**	**6.1**	**19.4**
	U	8.6	13.1	13.2	41.0	10.6	10.5	68.0	85.6	85.4	60.5	60.5	11.4
	O	45.3	43.1	43.0	23.1	43.3	43.3	15.4	6.5	6.6	12.8	12.8	5.4
	O	45.3	43.1	43.0	23.1	43.3	43.3	15.4	6.5	6.6	12.8	12.8	5.4
Peak 1 Transition 1.4: State 22	Cl	0.1	0.0	0.4	3.1	1.4	0.1	0.3	0.0	0.7	6.1	0.8	19.5
	Cl	0.1	0.0	0.4	3.1	1.4	0.1	0.3	0.0	0.7	6.1	0.8	19.5
	Cl	0.3	0.4	0.0	3.2	0.1	1.4	0.3	0.7	0.0	0.8	6.1	19.4
	Cl	0.3	0.4	0.0	3.2	0.1	1.4	0.3	0.7	0.0	0.8	6.1	19.4
	**U**	**9.7**	**13.6**	**13.6**	**41.4**	**10.5**	**10.5**	**68.9**	**85.3**	**85.3**	**60.6**	**60.6**	**11.4**
	**O**	**44.8**	**42.8**	**42.8**	**23.0**	**43.3**	**43.3**	**14.9**	**6.7**	**6.7**	**12.7**	**12.7**	**6.0**
	**O**	**44.8**	**42.8**	**42.8**	**23.0**	**43.3**	**43.3**	**14.9**	**6.7**	**6.7**	**12.7**	**12.7**	**6.0**
**Peak 2 Transition 2.1: State 36**	**Cl**	**0.1**	**0.0**	**0.4**	**3.1**	**1.4**	**0.1**	**0.3**	**0.1**	**0.6**	**6.1**	**0.9**	**19.2**
	**Cl**	**0.1**	**0.0**	**0.4**	**3.1**	**1.4**	**0.1**	**0.3**	**0.1**	**0.6**	**6.1**	**0.9**	**19.2**
	**Cl**	**0.3**	**0.4**	**0.0**	**3.2**	**0.1**	**1.4**	**0.3**	**0.6**	**0.1**	**0.9**	**6.1**	**19.2**
	**Cl**	**0.3**	**0.4**	**0.0**	**3.2**	**0.1**	**1.4**	**0.3**	**0.6**	**0.1**	**0.9**	**6.1**	**19.2**
	U	8.6	9.4	9.4	37.5	6.8	6.8	72.8	90.5	91.9	64.6	64.5	11.4
	O	45.3	44.9	44.9	26.2	45.5	45.5	12.8	4.1	3.2	10.6	10.5	5.4
	O	45.3	44.9	44.9	26.2	45.5	45.5	12.8	4.1	3.2	10.6	10.5	5.4
Peak 3 Transition 3.2: State 94	Cl	0.1	0.0	0.3	2.5	1.0	0.0	0.5	0.4	0.2	6.3	0.9	19.4
	Cl	0.1	0.0	0.3	2.5	1.0	0.0	0.5	0.4	0.2	6.3	0.9	19.4
	Cl	0.3	0.3	0.0	2.5	0.0	1.1	0.3	0.3	0.6	0.9	6.3	19.4
	Cl	0.3	0.3	0.0	2.5	0.0	1.1	0.3	0.3	0.6	0.9	6.3	19.4
	**U**	**8.5**	**9.5**	**9.4**	**37.2**	**6.7**	**6.9**	**72.6**	**91.2**	**92.1**	**65.0**	**63.6**	**11.4**
	**O**	**45.4**	**44.9**	**44.9**	**26.3**	**45.6**	**45.4**	**12.9**	**3.7**	**3.1**	**10.4**	**10.8**	**5.4**
	**O**	**45.4**	**44.9**	**44.9**	**26.3**	**45.6**	**45.4**	**12.9**	**3.7**	**3.1**	**10.4**	**10.8**	**5.4**
**Peak 3 Transition 3.6: State 120**	**Cl**	**0.1**	**0.0**	**0.3**	**2.6**	**1.0**	**0.0**	**0.4**	**0.6**	**0.2**	**6.3**	**0.9**	**19.5**
	**Cl**	**0.1**	**0.0**	**0.3**	**2.6**	**1.0**	**0.0**	**0.4**	**0.6**	**0.2**	**6.3**	**0.9**	**19.5**
	**Cl**	**0.3**	**0.4**	**0.0**	**2.6**	**0.0**	**1.1**	**0.4**	**0.2**	**0.7**	**0.9**	**6.6**	**19.4**
	**Cl**	**0.3**	**0.4**	**0.0**	**2.6**	**0.0**	**1.1**	**0.4**	**0.2**	**0.7**	**0.9**	**6.6**	**19.4**

Peak 3 Transition 3.7: State 132	U	8.7	9.8	10.4	38.6	7.1	6.9	72.1	88.8	88.8	64.2	65.0	11.5
	O	45.3	44.7	44.4	26.0	45.3	45.4	13.2	4.8	4.6	10.7	10.4	5.4
	O	45.3	44.7	44.4	26.0	45.3	45.4	13.2	4.8	4.6	10.7	10.4	5.4
	Cl	0.1	0.0	0.4	2.4	1.1	0.0	0.4	0.0	1.0	6.4	0.9	19.4
	Cl	0.1	0.0	0.4	2.4	1.1	0.0	0.4	0.0	1.0	6.4	0.9	19.4
	Cl	0.3	0.3	0.0	2.4	0.1	1.1	0.4	0.8	0.0	0.9	6.2	19.4
	Cl	0.3	0.3	0.0	2.4	0.1	1.1	0.4	0.8	0.0	0.9	6.2	19.4
Peak 3 Transition 3.8: State 133	U	8.7	9.8	9.3	38.0	6.8	6.9	72.7	89.0	89.3	64.8	64.1	11.5
	O	45.3	44.7	44.9	26.3	45.5	45.4	12.9	4.7	4.5	10.5	10.6	5.4
	O	45.3	44.7	44.9	26.3	45.5	45.4	12.9	4.7	4.5	10.5	10.6	5.4
	Cl	0.1	0.0	0.3	2.3	1.0	0.1	0.4	0.0	0.8	6.3	0.9	19.4
	Cl	0.1	0.0	0.3	2.3	1.0	0.1	0.4	0.0	0.8	6.3	0.9	19.4
	Cl	0.3	0.4	0.0	2.3	0.0	1.1	0.4	0.8	0.0	0.9	6.5	19.4
	Cl	0.3	0.4	0.0	2.3	0.0	1.1	0.4	0.8	0.0	0.9	6.5	19.4
Peak 4 Transition 4.1: State 2318	U	9.6	9.2	9.2	36.7	6.4	6.4	73.2	89.6	89.6	64.6	64.6	11.5
	O	44.8	45.0	45.0	25.0	45.8	45.7	12.7	4.5	4.5	10.2	10.2	6.0
	O	44.8	45.0	45.0	25.0	45.8	45.7	12.7	4.5	4.5	10.2	10.2	6.0
	Cl	0.1	0.0	0.4	3.3	1.0	0.0	0.4	0.1	0.7	6.6	0.9	19.2
	Cl	0.1	0.0	0.4	3.3	1.0	0.0	0.4	0.1	0.7	6.6	0.9	19.2
	Cl	0.3	0.4	0.0	3.4	0.0	1.0	0.4	0.7	0.1	0.9	6.6	19.1
	Cl	0.3	0.4	0.0	3.4	0.0	1.0	0.4	0.7	0.1	0.9	6.6	19.1
Peak 4 Transition 4.2: State 2435	U	8.6	16.2	16.2	41.7	8.2	8.2	67.0	82.1	82.1	61.9	61.9	11.3
	O	45.3	41.5	41.4	22.2	44.6	44.6	15.7	8.3	8.3	11.6	11.6	4.1
	O	45.3	41.5	41.4	22.2	44.6	44.6	15.7	8.3	8.3	11.6	11.6	4.1
	Cl	0.1	0.0	0.4	3.5	1.2	0.1	0.4	0.1	0.6	6.5	0.9	20.1
	Cl	0.1	0.0	0.4	3.5	1.2	0.1	0.4	0.1	0.6	6.5	0.9	20.1
	Cl	0.3	0.4	0.0	3.5	0.1	1.2	0.4	0.6	0.1	0.9	6.5	20.1
	Cl	0.3	0.4	0.0	3.5	0.1	1.2	0.4	0.6	0.1	0.9	6.5	20.1

Table S15: Atoms in molecules (AIM) percentage composition analysis for $Cs_2UO_2Cl_4$ SONOs in RAS(SD) states. Bolded values identify data chosen to be represented in the main text.

		σg	πu		σu	πg		σu*	πu*		πg*		σg*
	U	14.1	19.1	19.2	47.8	12.2	12.0	58.9	77.6	77.7	52.7	52.4	54.1
	O	42.7	39.9	39.9	25.2	42.7	42.7	20.5	10.8	10.7	19.8	19.7	21.6
	O	42.7	39.9	39.9	25.2	42.7	42.7	20.5	10.8	10.7	19.8	19.7	21.6
Peak0: Transition 0: Ground-State	Cl	0.1	0.3	0.3	0.4	0.6	0.6	0.0	0.2	0.2	1.9	2.0	0.7
	Cl	0.1	0.3	0.3	0.4	0.6	0.6	0.0	0.2	0.2	1.9	2.0	0.7
	Cl	0.1	0.3	0.3	0.4	0.6	0.6	0.0	0.2	0.2	1.9	2.0	0.7
	Cl	0.1	0.3	0.3	0.4	0.6	0.6	0.0	0.2	0.2	1.9	2.0	0.7
	**U**	**9.6**	**12.3**	**12.6**	**40.6**	**12.1**	**11.9**	**68.1**	**85.6**	**85.4**	**60.0**	**59.6**	**9.6**
	**O**	**41.2**	**42.3**	**42.2**	**22.9**	**40.9**	**41.0**	**15.3**	**6.3**	**6.4**	**12.8**	**12.6**	**5.4**
	**O**	**41.2**	**42.3**	**42.2**	**22.9**	**40.9**	**41.0**	**15.3**	**6.3**	**6.4**	**12.8**	**12.6**	**5.4**
**Peak 1 Transition 1.4: State 24**	**Cl**	**2.0**	**0.8**	**0.7**	**3.4**	**1.5**	**1.5**	**0.3**	**0.4**	**0.5**	**3.6**	**3.8**	**19.9**
	**Cl**	**2.0**	**0.8**	**0.7**	**3.4**	**1.5**	**1.5**	**0.3**	**0.4**	**0.5**	**3.6**	**3.8**	**19.9**
	**Cl**	**2.0**	**0.8**	**0.7**	**3.4**	**1.5**	**1.5**	**0.3**	**0.4**	**0.5**	**3.6**	**3.8**	**19.9**
	**Cl**	**2.0**	**0.8**	**0.7**	**3.4**	**1.5**	**1.5**	**0.3**	**0.4**	**0.5**	**3.6**	**3.8**	**19.9**
	**U**	**10.7**	**12.9**	**13.0**	**41.1**	**12.2**	**12.1**	**68.6**	**85.2**	**85.2**	**59.9**	**59.4**	**9.6**
	**O**	**40.7**	**42.0**	**42.0**	**22.3**	**40.9**	**40.9**	**15.0**	**6.5**	**6.5**	**12.8**	**12.7**	**6.1**
	**O**	**40.7**	**42.0**	**42.0**	**22.3**	**40.9**	**40.9**	**15.0**	**6.5**	**6.5**	**12.8**	**12.7**	**6.1**
**Peak 2 Transition 2.2: State 29**	**Cl**	**2.0**	**0.8**	**0.7**	**3.6**	**1.5**	**1.5**	**0.3**	**0.4**	**0.4**	**3.6**	**3.8**	**19.5**
	**Cl**	**2.0**	**0.8**	**0.7**	**3.6**	**1.5**	**1.5**	**0.3**	**0.4**	**0.4**	**3.6**	**3.8**	**19.5**
	**Cl**	**2.0**	**0.8**	**0.7**	**3.6**	**1.5**	**1.5**	**0.3**	**0.4**	**0.4**	**3.6**	**3.8**	**19.5**
	**Cl**	**2.0**	**0.8**	**0.7**	**3.6**	**1.5**	**1.5**	**0.3**	**0.4**	**0.4**	**3.6**	**3.8**	**19.5**
	**U**	**9.5**	**8.8**	**9.1**	**38.0**	**8.1**	**7.9**	**72.5**	**89.2**	**88.9**	**64.0**	**64.0**	**9.7**
	**O**	**41.3**	**44.0**	**43.9**	**25.9**	**43.0**	**43.1**	**13.0**	**4.7**	**4.8**	**10.7**	**10.5**	**5.4**
	**O**	**41.3**	**44.0**	**43.9**	**25.9**	**43.0**	**43.1**	**13.0**	**4.7**	**4.8**	**10.7**	**10.5**	**5.4**
**Peak 3 Transition 3.1: State 92**	**Cl**	**2.0**	**0.8**	**0.8**	**2.6**	**1.5**	**1.5**	**0.4**	**0.4**	**0.4**	**3.6**	**3.7**	**19.9**
	**Cl**	**2.0**	**0.8**	**0.8**	**2.6**	**1.5**	**1.5**	**0.4**	**0.4**	**0.4**	**3.6**	**3.7**	**19.9**
	**Cl**	**2.0**	**0.8**	**0.8**	**2.6**	**1.5**	**1.5**	**0.4**	**0.4**	**0.4**	**3.6**	**3.7**	**19.9**
	**Cl**	**2.0**	**0.8**	**0.8**	**2.6**	**1.5**	**1.5**	**0.4**	**0.4**	**0.4**	**3.6**	**3.7**	**19.9**
	U	9.5	8.3	8.2	36.3	7.7	7.8	73.1	91.4	92.8	64.5	63.1	9.6
	O	41.3	44.2	44.3	26.2	43.2	43.1	12.6	2.5	1.6	10.5	10.6	5.4
	O	41.3	44.2	44.3	26.2	43.2	43.1	12.6	2.5	1.6	10.5	10.6	5.4
Peak 3 Transition 3.2: State 111	Cl	2.0	0.8	0.8	2.9	1.5	1.5	0.5	0.9	1.0	3.6	3.9	19.9
	Cl	2.0	0.8	0.8	2.9	1.5	1.5	0.5	0.9	1.0	3.6	3.9	19.9
	Cl	2.0	0.8	0.8	2.9	1.5	1.5	0.5	0.9	1.0	3.6	3.9	19.9
	Cl	2.0	0.8	0.8	2.9	1.5	1.5	0.5	0.9	1.0	3.6	3.9	19.9

7. Oscillator Strengths versus Anti-Bonding Orbital Oxygen character:

Table S16. Total oxygen percentage in the anti-bonding orbitals assigned to key core-excitations for $[UO_2]^{2+}$ RAS(SD) simulation obtained through various methods. Percentages are averages to account for the two orbitals in each set of π_u^* and π_g^*. Values are used to plot figure S11. Values in brackets are oxygen p-character. AIM $[UO_2]^{2+}$ data taken from simulations reported in ref 1.

**Method**	**Peak 1 Transition 1.4 State 24 π_u^***	**Peak 2 Transition 2.5 State 99 σ_u^***	**Peak 3 Transition 3.8 State 395 π_g^***
AIM	14.8	31.8	25.4
Ros-Schuit (SCPA)	11.5 (11.5)	31.1 (25.3)	26.0 (25.9)
Mulliken	13.5 (13.6)	31.9 (30.4)	5.4 (5.1)
Stout-Politzer	22.6 (22.6)	53.5 (38.4)	42.0 (41.9)
Hirshfeld	15.1	31.2	23.7
Becke	13.3	27.7	18.3

Table S17. Oxygen compositions from table S16 normalised and used to plot main text figure 4. Values in brackets are the normalised oxygen p-character from table S16. AIM $[UO_2]^{2+}$ data taken from simulations reported in ref 1.

**Method**	**Peak 1 Transition 1.4 State 24 π_u^***	**Peak 2 Transition 2.5 State 99 σ_u^***	**Peak 3 Transition 3.8 State 395 π_g^***
AIM	0.5	1.0	0.8
Ros-Schuit (SCPA)	0.4 (0.4)	1.0 (1.0)	0.8 (1.0)
Mulliken	0.4 (0.4)	1.0 (1.0)	0.2 (0.2)
Stout-Politzer	0.4 (0.5)	1.0 (0.9)	0.8 (1.0)
Hirshfeld	0.5	1.0	0.8
Becke	0.5	1.0	0.7

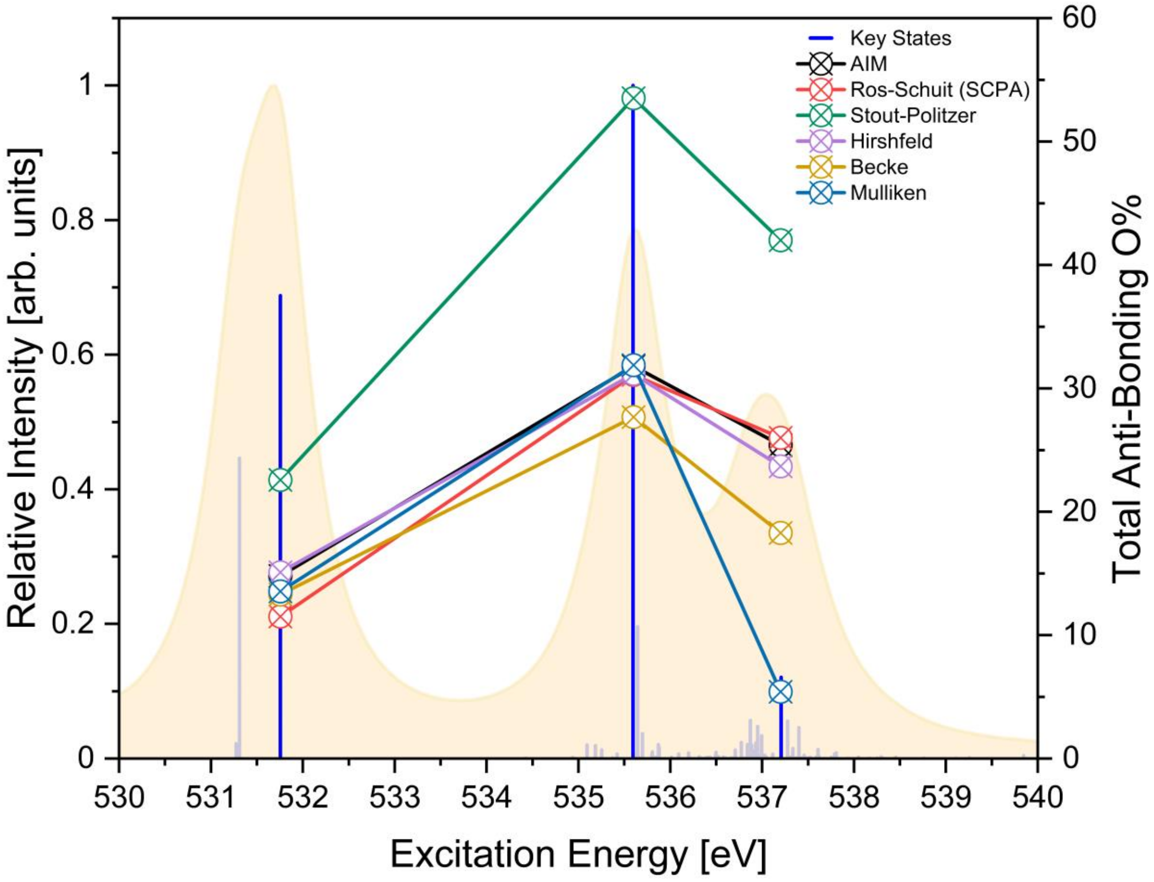

Figure S11: Plot showing total oxygen percentage to anti-bonding orbitals for key transitions in the RAS(SD) $[UO_2]^{2+}$ simulations. Oxygen percentages are taken from table S16 and plotted at the energy position of the transition. Both the profile and transition stick intensities have been normalised with respect to the global maximum value.

(a) (b)

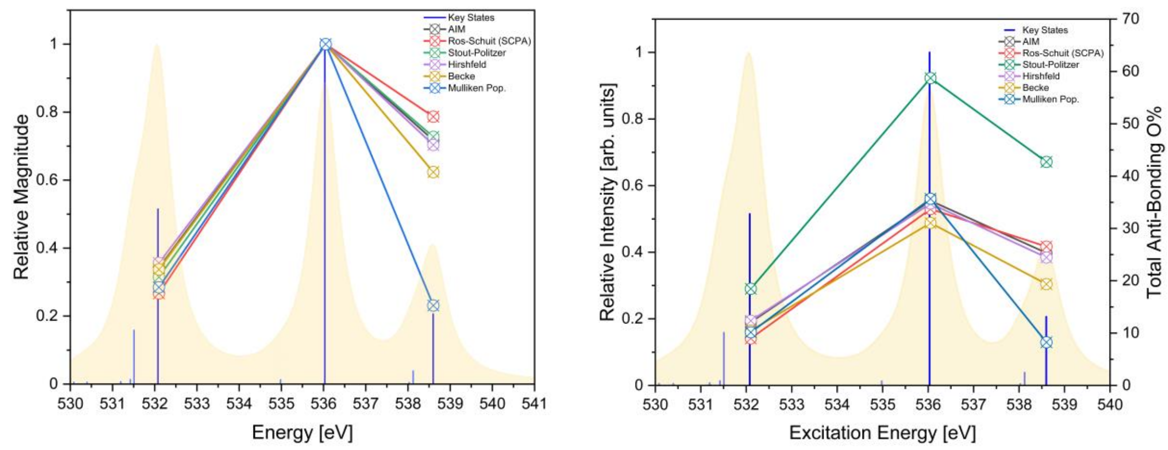

Figure S12: (a) Plot showing the relative and normalised trends in total oxygen percentage to the anti-bonding orbital for key RAS(S) core-ESs. (b) Plot showing the total oxygen composition to anti-bonding orbitals for key RAS(S) core-ESs.

8. Structure Coordinates:

D_{2h} structure models were built with reference to $Cs_2UO_2Cl_4$ crystal in ref [4] used by Denning in the experimental reference [2] and based on the CIF file in the Crystallography Open Database [https://www.crystallography.net/cod/]. COD ID: 2020346.

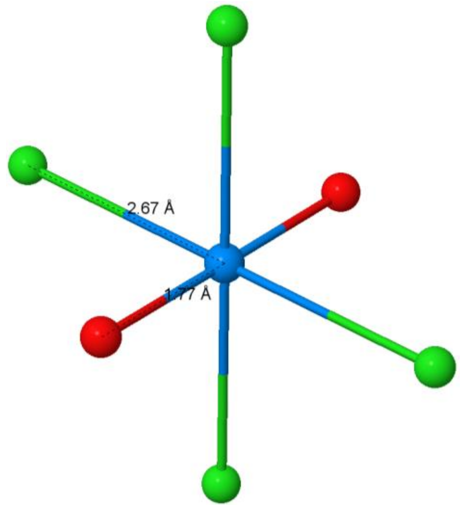

Figure S13: U-O (1.77Å) and U-Cl (2.67Å) bond lengths in the $[UO_2Cl_4]^{2-}$ structure model.

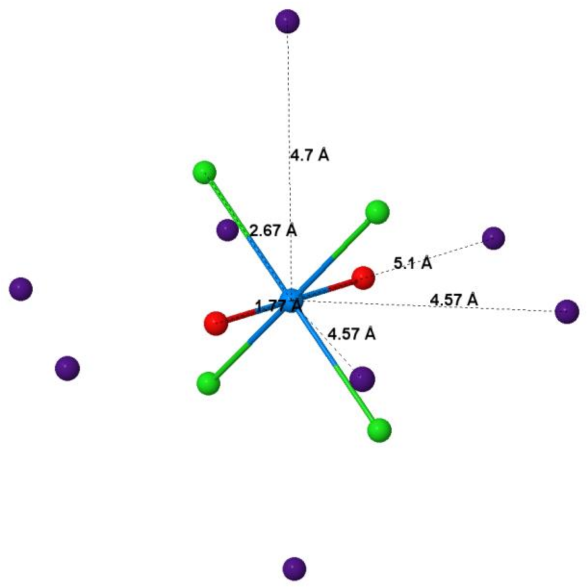

Figure S14: Bond lengths (Å) for the $Cs_2UO_2Cl_4$ structure model.

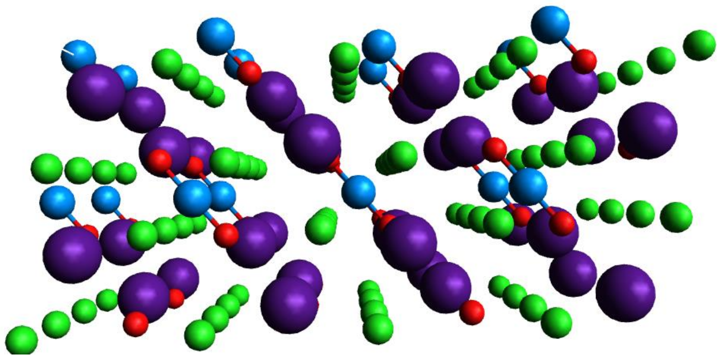

Figure S15: Uranyl super cell built from CIF file obtained from the Crystallography Open Database (COD ID: 2020346) utilized to inform $Cs_2UO_2Cl_4$ model. [4]

Table S18: $[UO_2]^{2+}$ D_{2h} X,Y,Z atomic coordinates.

Center	Label	x	y	z
1	U	0.00	0.00	0.00
2	O	0.00	0.00	1.77
3	O	0.00	0.00	-1.77

Table S19: $[UO_2Cl_4]^{2-}$ D_{2h} X,Y,Z atomic coordinates.

Center	Label	x	y	z
1	U	0.00	0.00	0.00
2	O	0.00	0.00	1.77
3	O	0.00	0.00	-1.77
4	CL	2.67	0.00	0.00
5	CL	-2.67	0.00	0.00
6	CL	0.00	2.67	0.00
7	CL	0.00	-2.67	0.00

Table S20: $Cs_2UO_2Cl_4$ D_{2h} X,Y,Z atomic coordinates.

Center	Label	x	y	z
1	U	0.00	0.00	0.00
2	O	0.00	0.00	1.77
3	O	0.00	0.00	-1.77
4	CL	1.84	1.94	0.00
5	CL	-1.84	-1.94	0.00
6	CL	-1.84	1.94	0.00
7	CL	1.84	-1.94	0.00
8	Cs	4.70	0.00	0.00
9	Cs	-4.70	0.00	0.00
10	Cs	0.00	0.00	5.10
11	Cs	0.00	0.00	-5.10
12	Cs	0.00	3.85	2.47
13	Cs	0.00	-3.85	2.47
14	Cs	0.00	3.85	-2.47
15	Cs	0.00	-3.85	-2.47

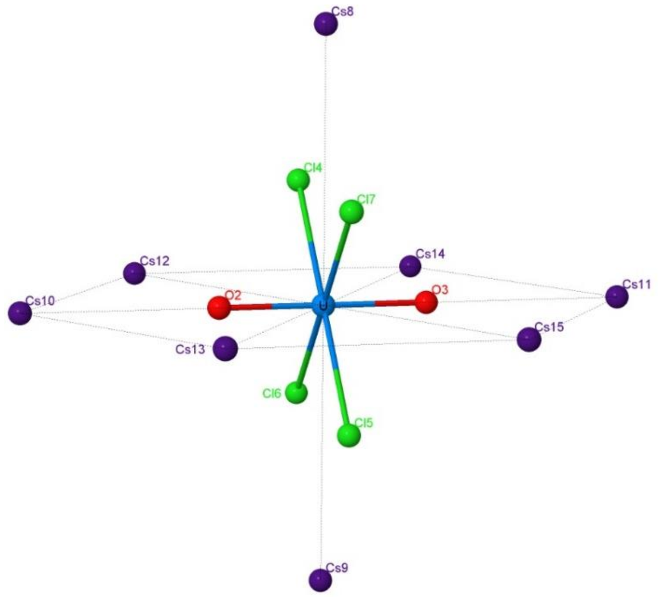

Figure S16: $Cs_2UO_2Cl_4$ structure showing atomic labels utilized in tables S20/S21.

Table S21: Table of structure parameters for the experimental $Cs_2UO_2Cl_4$ system and the D_{2h} structure.

Measurement	D_{2h} $Cs_2UO_2Cl_4$ Model	Experimental $Cs_2UO_2Cl_4$
U-O2	1.77	1.77
U-Cl4	2.67	2.67
U-Cs13	4.57	4.58
U-Cs15	4.57	4.58
U-Cs11	5.1	5.1
U-Cs8	4.7	4.7
Cl4-U-Cl7	93.0	92.9
Cl7-U-Cl5	87.0	87.1
Cs10-U-Cs8	90	72.7
Cs13-U-Cs15	65.4	65.4
Cs10-U-Cs13	57.3	57.3

9. Peak Broadenings:

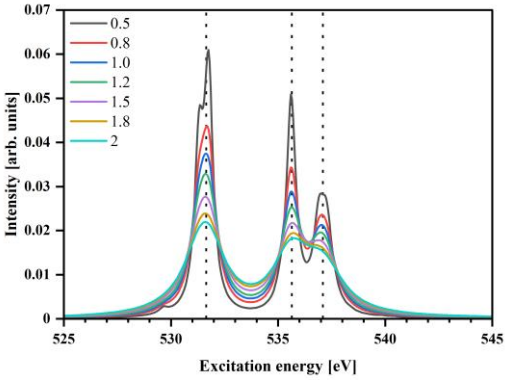

Figure S17: Lorentzian broadening of $[UO_2]^{2+}$ RAS(SD) transitions.

10. References:

[1] Kurtis Stanistreet-Welsh and Andrew Kerridge, *Phys. Chem. Chem. Phys.*, 2023,**25**, 23753-23760. [https://doi.org/10.1039/D3CP03149G]

[2] R. G. Denning, J. C. Green, T. E. Hutchings, C. Dallera, A. Tagliaferri, K. Giarda, N. B. Brookes and L. Braicovich, *J Chem Phys*, 2002, **117**, 8008–8020. [https://doi.org/10.1063/1.1510445]

[3] C. Fillaux, D. Guillaumont, J.-C. Berthet, R. Copping, D. K. Shuh, T. Tyliszczak and C. Den Auwer, *Phys. Chem. Chem. Phys.*, 2010, **12**, 14253–14262. [https://doi.org/10.1039/C0CP00386G]

[4] The crystal structure of dicaesium tetrachlorodioxouranium(VI), D. Hall, A. D. Rae and T. N. Waters, *Acta Cryst.* ,1966. **20**, 160-162. [https://doi.org/10.1107/S0365110X66000355]
